# Augmenting MEK inhibitor efficacy in BRAF wild-type melanoma: synergistic effects of disulfiram combination therapy

**DOI:** 10.1186/s13046-023-02941-5

**Published:** 2024-01-23

**Authors:** Francisco Meraz-Torres, Heike Niessner, Sarah Plöger, Simon Riel, Barbara Schörg, Nicolas Casadei, Manfred Kneilling, Martin Schaller, Lukas Flatz, Boris Macek, Thomas Eigentler, Olaf Rieß, Claus Garbe, Teresa Amaral, Tobias Sinnberg

**Affiliations:** 1 Department of Dermatology, Tübingen University Hospital, Tübingen, Germany; 2 Cluster of Excellence iFIT (EXC 2180) Image Guided and Functionally Instructed Tumor Therapies, University Hospital Tübingen, Tübingen, 72076 Germany; 3 Department of Preclinical Imaging and Radiopharmacy, Laboratory for Preclinical Imaging and Imaging Technology of the Werner Siemens-Foundation, University of Tübingen, Tübingen, 72076 Germany; 4 NGS Competence Center Tübingen, Institute of Medical Genetics and Applied Genomics, University of Tübingen, Tübingen, Germany; 5 Proteome Center Tübingen, University of Tübingen, Tübingen, Germany; 6 Department of Dermatology, Venereology and Allergology, Charité - Universitätsmedizin Berlin, Charitéplatz 1, Berlin, 10117 Germany

**Keywords:** Melanoma, BRAF, Disulfiram, Trametinib, MEK inhibitor, ER stress

## Abstract

**Background:**

MEK inhibitors (MEKi) were shown to be clinically insufficiently effective in patients suffering from BRAF wild-type (BRAF WT) melanoma, even if the MAPK pathway was constitutively activated due to mutations in NRAS or NF-1. Thus, novel combinations are needed to increase the efficacy and duration of response to MEKi in BRAF WT melanoma. Disulfiram and its metabolite diethyldithiocarbamate are known to have antitumor effects related to cellular stress, and induction of endoplasmic reticulum (ER) stress was found to synergize with MEK inhibitors in NRAS-mutated melanoma cells. Therefore, we investigated the combination of both therapeutics to test their effects on BRAF-WT melanoma cells and compared them with monotherapy using the MEKi trametinib.

**Methods:**

The effects of combined therapy with disulfiram or its metabolite diethyldithiocarbamate and the MEKi trametinib were evaluated in a series of BRAF-WT melanoma cell lines by measuring cell viability and apoptosis induction. Cytotoxicity was additionally assessed in 3D spheroids, ex vivo melanoma slice cultures, and in vivo xenograft mouse models. The response of melanoma cells to treatment was studied at the RNA and protein levels to decipher the mode of action. Intracellular and intratumoral copper measurements were performed to investigate the role of copper ions in the antitumor cytotoxicity of disulfiram and its combination with the MEKi.

**Results:**

Diethyldithiocarbamate enhanced trametinib-induced cytotoxicity and apoptosis induction in 2D and 3D melanoma culture models. Mechanistically, copper-dependent induction of oxidative stress and ER stress led to Janus kinase (JNK)-mediated apoptosis in melanoma cells. This mechanism was also detectable in patient-derived xenograft melanoma models and resulted in a significantly improved therapeutic effect compared to monotherapy with the MEKi trametinib.

**Conclusions:**

Disulfiram and its metabolite represent an attractive pharmaceutical approach to induce ER stress in melanoma cells that potentiates the antitumor effect of MEK inhibition and may be an interesting candidate for combination therapy of BRAF WT melanoma.

**Supplementary Information:**

The online version contains supplementary material available at 10.1186/s13046-023-02941-5.

## Introduction

In recent years, game-changing therapies have significantly improved the overall survival of melanoma patients. In metastatic melanoma in general, but particularly in BRAF wild-type melanoma, the approved immunotherapies anti-PD1 [1, 2], anti-CTLA-4 [3] and, since 2022, anti-LAG3 [4] are being used. Although anti-PD1-based combination therapies in particular show remarkable response rates and durable responses [5], primary resistance [6–8] and toxicities [9, 10] are causes of treatment failure and force therapy changes. However, alternatives are sparse and essentially include enrollment in clinical trials such as TIL therapy [11] or fecal microbiota transplantation [12, 13], as well as chemotherapy. Therefore, therapy resistance remains a challenging clinical problem for up to 50% of patients treated with immunotherapy. In addition, no targeted therapy has been approved for BRAF-WT melanomas, despite the fact that this subgroup accounts for the majority of cases, often has activation of the MAPK pathway [14], and has an aggressive cell biology with an unfavorable prognosis [15, 16].

Beyond the BRAF (V600) hotspot mutations, which occur in approximately 40% of all cutaneous melanomas, other mutations in genes such as NRAS and NF1 cause constitutive hyperactivation of the mitogen-activated protein kinase (MAPK) pathway in another 30-35% of the cases [17]. Therefore, therapy with MEKi seems to be a logical consequence for BRAF WT melanomas with a mutation-activated MAPK pathway [18, 19]. Trametinib (GSK1120212) is a reversible allosteric inhibitor of MEK1 and MEK2. In a phase I trial, it was initially tested on 206 patients, showing effective inhibition of MAPK signaling and proliferation in biopsies that were taken under treatment [20]. However, although the response rate was 40% in treatment-naïve patients with known BRAF mutations, it was only 10% for the cases with BRAF-WT melanoma and none of seven patients with NRAS-mutated melanoma showed a response. Therefore, further phase II and III studies evaluating trametinib against melanoma successfully focused on BRAF-mutated melanoma [21–23]. The phase 3 NEMO trial evaluated binimetinib, another MEKi, versus dacarbazine chemotherapy in patients with advanced NRAS-mutated melanoma [24]. The median PFS improved to 2.8 months with binimetinib versus 1.5 months with dacarbazine. The median overall survival (OS) reached 11.0 months with the MEKi versus 10.1 months with the chemotherapy and the overall response rate of binimetinib monotherapy remained poor (15%) but still higher than in the dacarbazine group (7%). Given the good results obtained with BRAF inhibitors for the treatment of BRAF(V600E/K) melanoma, this result was disappointing, and no approval was granted [24]. So far, three different MEKi namely trametinib, cobimetinib and binimetinib are clinically used against BRAF-mutated melanomas usually in combination with their corresponding BRAF inhibitor. Among these, trametinib was shown to have the lowest IC50 and highest inhibitory potential. In a comparative analysis of various MEK inhibitors, trametinib demonstrated potent inhibitory activity against unphosphorylated and phosphorylated MEK, with an IC50 value of 0.42 nM and 11 nM, respectively [25]. Concordantly, trametinib showed excellent performance in combination with different BRAFi when compared with the other approved MEKi [26]. A recent retrospective, multi-center study evaluated the clinical course of patients with NRAS mutated stage IV melanoma treated with MEKi and at least one prior line of treatment. Trametinib showed the best results, but the overall response rate across all MEKi remained low at 18.2% [27]. Due to the high costs associated with drug development, a promising strategy to improve the efficacy of MEKi in BRAF WT melanoma is to repurpose an already approved drug and combine it with a generally effective MEKi like trametinib [28–30]. Disulfiram (DSF), a U.S. Food and Drug Administration (FDA)-approved inhibitor of the aldehyde dehydrogenase (ALDH) for the treatment of alcohol abuse, has emerged as a promising candidate for combinations with anticancer drugs. It has been used clinically for decades and was shown to inhibit tumor growth as a side effect [31–34]. Accordingly, the pharmacokinetics, tolerability and safety (median lethal dose [LD50] of 8.6 g/kg) are well known [32, 35]. In the body, DFS is rapidly metabolized by its reduction into two molecules of diethyldithiocarbamate (ET [32, 36]. ET was shown to chelate bivalent metal ions, particularly copper II (Cu^2+^), forming a stable complex (CuET) with its thiol groups in the blood. This complex showed measurable antitumor activity in different cancer models, including melanoma [35, 37–39]. Studies suggest that the anticancer effect of DSF is not related to the inhibition of ALDH, but is rather due to the the copper-containing metabolite CuET [34].

In accordance with its preclinical antitumor effects, DSF was also tested clinically in several cancers: Against stage IV melanoma as monotherapy (NCT00256230) and in combination with arsenic trioxide (NCT00571116) or chelated zinc (Zn^2+^) (NCT02101008); in combination with 5-fluorouracil against glioblastoma [40, 41], non-small cell lung cancer [42] and colon carcinoma [43, 44]; and in combination with chemotherapy against breast cancer [45, 46]. Of note, lower cancer-specific mortality due to colorectal, prostate, and breast cancer was observed in Danish patients taking DSF for the treatment of alcohol abuse [35, 47].

These data led to our hypothesis that DSF has an anticancer effect on BRAF WT melanoma cells and that its combination with the MEKi trametinib results in potent killing of melanoma cells. To verify this, we used the active metabolite of DSF in its copper-bound form CuET in our preclinical in vitro studies and combined it with the MEKi trametinib to treat BRAF-WT melanoma cells. For in vivo experiments in mice, we used DSF because it is rapidly converted to CuET in the blood.

## Methods

### Chemicals and inhibitors

Stock solutions were prepared in dimethyl sulfoxide (Applichem): MEK1/2 inhibitor trametinib (1 mM; biomol #1187431), disulfiram (1 mM; Sigma Aldrich #97-77-8), ammonium diethyldithiocarbamate (1 mM; Sigma Aldrich #359548), ammonium tetrathiomolybdate (10 mM; Sigma Aldrich #15060), pan-caspase inhibitor Z-VAD-FMK (20 µM; InvivoGen #tlrl-vad), and JUN N-terminal kinase inhibitor SP600125 (100 mM, InvivoGen #tlrl-sp60). Stock solutions were prepared in aqueous buffer: copper (II) D-gluconate (1 mM, Sigma Aldrich #344419) in PBS and N-acetyl-L-cysteine (10 mM; Sigma Aldrich #A7250) in RPMI medium.

### Melanoma cell lines and patient-derived melanoma cells

All melanoma cells were cultured in RPMI 1640 medium (Gibco) with 10% fetal bovine serum (Sigma Aldrich) and 1% penicillin and streptomycin (Gibco) [48]. The melanoma cell lines SKMEL23 and SKMEL113 were kindly provided by Prof. Ralf Gutzmer (Medizinische Hochschule Hannover, Germany). 451Lu, MEL1617, WM793 and WM1366 melanoma cell lines were kindly provided by Prof. Meenhard Herlyn (Wistar Institute, Philadelphia, USA). A375 and MELG361 cells were purchased from the American Type Culture Collection (ATCC). Patient-derived short-term cultures (PDSC) (TÜMEL1, TÜMEL19, TÜMEL28, TÜMEL61, TÜMEL62-1, TÜMEL78, TÜMEL96, TÜMEL110, TÜMEL119, TÜMEL123-1, TÜMEL173, TÜMEL176) were either isolated directly from metastatic melanomas or were first expanded as PDX in NSG mice before cells were isolated out of the PDX tumor. Tissue was cut into small pieces and incubated in an enzymatic solution (HBBS without Ca^2+^ and Mg^2+^, 0.05% collagenase, 0.1% hyaluronidase, 1.25 U/ml dispase) at 37°C for 1 hour to obtain a single-cell suspension, which was further cultured under standard conditions.

### MUH cell viability assay

Cell viability was measured using the 4-methylumbelliferyl heptanoate (MUH) assay, as formerly described using 2,500 cells per well [49, 50]. The substrate MUH is converted by lipases of living cells into 4-methylumbelliferone, which can be measured as a surrogate for cell viability or biomass. The effects of proliferation, metabolism, cell survival or cell death are summarized in this assay under the term viability or biomass. Cells were treated in hexaplicates for 72 hours, incubated for 1 hour at 37°C with 100 μg/mL MUH and fluorescence (λ_ex_=355 nm/λ_em_=460 nm) was measured with a Tristar microplate reader (Berthold Technologies). Dose-response analysis was done using GraphPad Prism and a sigmoidal 4-parameter logistics regression model.

### Clonogenic growth assay

Five hundred cells were seeded per cavity in 12-well plates and cultivated for 24 hours in standard growth medium. Cells were treated in triplicates for 14 days. The culture medium was changed every third day with corresponding treatments. Cells were fixed with 4% paraformaldehyde for 15 minutes and stained for 2 hours with a 3% crystal violet solution (Sigma-Aldrich). Plates were scanned and colonies were counted.

### Spheroid assay

Hanging drops of melanoma cells (250 cells/ 25 μl-drop) in standard growth medium were cultivated for 7 days to induce spheroid formation. Ten spheroids were embedded in 1 ml medium with 1.2 mg/mL collagen I (Corning) in a 12-well plate. Light microscopic images were taken daily from day 0 to day 6. ImageJ software was used to quantify the spheroid diameter and size. At day 7, spheroids were stained with 8 µg/mL propidium iodide for cell death (Sigma Aldrich) and 2 µM calcein-AM (Biolegend) for viability and analyzed with an Axioplan 200 fluorescence microscope (Zeiss).

### Cell cycle analysis

3x10^5^ cells/cavity were seeded into 6-well plates and treated the next day for 24, 48 and 72 hours in triplicates. Cells were harvested and permeabilized with 70% ice-cold ethanol and stained in PBS containing 50 µg/mL propidium iodide (Sigma-Aldrich) and 100 μg/mL RNaseA (AppliChem). Cell cycle distribution was analyzed by an LSRII flow cytometer (BD Biosciences) [49].

### Immunoblot analysis

Lysates were prepared using RIPA buffer with protease and phosphatase inhibitors and cleared by centrifugation for 20 minutes at 13,000 rpm. Proteins were separated using 10% polyacrylamide gels and blotted onto PVDF membranes, as described previously [49, 51]. The following primary antibodies and dilutions were used: anti-ATOX1 (1:250, Santa Cruz, sc-398742), anti-phospho-ERK1/2 (1:1000, CST,#4370), anti-ERK1/2 (1:1000, CST, #4696), anti-β-Actin (1:1000, CST,#4696), anti-phospho-H2A.X (Ser139) (1:500, CST, #9718), anti α-Tubulin (1:1000, CST, #2144 ), anti-LaminB (c-20) (1:200, Santa Cruz,#sc-6216), anti-Caspase-3 (1:1000, CST, #9668), anti-cleaved Caspase-3 (1:1000, CST, #9664), anti-Bad (1:1000, CST,#9292), anti-BAX (1:250,BD Biosciences, #556467), anti-BCL-2 (1:250, Santa Cruz,#sc-509), anti-BIM (C34C5)(1:1000, CST, #2933), anti-PARP (1:1000,CST, #9532), anti-Cleaved PARP-3 (Asp214) (#5625), anti-JUN (1:1000,CST, #9165), Anti-SAPK/JNK1/2/3 (1:1000, CST, #9252), anti-PJNK1/2/3 (1:250,Santa Cruz, #sc-6254), anti- p21 Waf1/Cip1 (1:1000,CST, #2946), anti-p53 (DO-1) (1:500, Santa Cruz, #sc-126), anti-CHOP (L63F7) (1:500, Cell Signaling, #2895), anti-ATF-4 (D4B8) (1:500, CST, #11815), anti-p8 (1:250, abcam, #ab6028). Peroxidase-conjugated secondary antibodies (Cell Signaling) and ECL or ECLplus as substrate (Thermo Scientific) were used for chemiluminescent detection.

### Immunofluorescence staining

Tissue was fixed in PBS-buffered 4% formaldehyde and embedded in paraffin (FFPE). Subsequently, 3 µm tissue slices were prepared for staining with antibodies specific for JUN (CST, #9165; 1:200 dilution) and phospho-ERK1/2 (CST, #4370; 1:100 dilution). Secondary antibodies (Cy^TM^3-conjugated AffiniPure F(ab’)_2_; Jackson ImmunoResearch #715-166-150 dilution 1:250, and Cy^TM^5-conjugated AffiniPure (ab’)_2_; Jackson ImmunoResearch #711-176-152 dilution 1:250) were used. Nuclei were stained with YO-PRO^TM^-1 (Thermo Fisher; #491/509) at a dilution of 1:250.

For the detection of copper ions in melanoma cells, 3×10^4^ cells/well were seeded per cavity in 12-well plates, treated for 6 hours and stained with 5 µM CopperGreen® (Goryo Chemical) plus DAPI (Invitrogen). Samples were analyzed using an LSM 800 confocal laser-scanning microscope (Zeiss).

### Small interfering RNA (siRNA) transfection

siRNAs (siGENOME human, Riboxx) were transfected using Lipofectamine RNAiMAX and OptiMEM medium (both Thermo Fisher Scientific) according to the manufacturer’s protocol and as described previously [50]. Guide and passenger sequences that were used are presented in Supplementary Table 2.

### RNA isolation and real time-qPCR

Total RNA was isolated using NucleoSpin RNA spin columns (Machery-Nagel) and reverse transcribed with the Maxima First Strand cDNA Synthesis Kit (Thermo Scientific), and quantitative real-time qPCR analysis was performed using GoTaq® qPCR Master Mix (Promega). The primers that were used are listed in Supplementary Table 1. Quantification of gene expression was carried out with a Lightcycler96 (Roche), normalized to the expression of TBP and ACTB.

### Copper uptake assay

Two million melanoma cells were seeded in a T75 flask (Sarstedt) and treated the following day for 6 hours. The cells were harvested, washed two times with ice-cold PBS and resuspended in 1 mL of 333 nM HCl. After incubation at 60°C for 3 hours, 750 μL of the suspension was added to 250 μL 30% trichloroacetic acid (Merck) and centrifuged at 13,000 xg for 5 min. 500 μL of the supernatant was added into a new tube, and 100 μL of 2 mM L-dehydroascorbic acid (Sigma Aldrich) was added. Finally, 400 μL of 150 μM bicinchoninic acid disodium (Sigma Aldrich), 900 mM NaOH (Merck) and 600 mM HEPES sodium (AppliChem) was added. After 24 hours of incubation at 37 °C, the absorbance was measured with a photometer (Bio-Rad) at λ=354 nm and λ=560 nm. A Cu^2+^ standard curve from 100 μM to 0 μM in 333 mM HCl was used to calculate the copper concentration.

Subcellular fractionation of 2x10^6^ melanoma cells was done in 500 μL of fractionation buffer (20 mM HEPES sodium pH 7.4, AppliChem; 10 mM KCl, Sigma Aldrich; 2 mM MgCl_2_, Sigma Aldrich; 1 mM EDTA, (Carl Roth), 1 mM DTT (Sigma Aldrich). Cells were passed ten times through a 27-gauge needle, incubated for 20 min on ice and centrifuged for 5 min at 720 xg at 4°C to pellet nuclei. Further fractionation was performed by passing the solution repeatedly through a 25-gauge needle followed by 5 minutes of centrifugation at 10,000 xg and 4°C, resulting in pelleted mitochondria. Cytoplasm-membrane fractions remained in the supernatant. Next, the copper content was analyzed as described above.

### Patient-derived xenografts

*A total of* 1×10^6^ BRAF WT melanoma cells (TÜMEL62-1, TÜMEL110 and TÜMEL173 representing metastases from three different patients) were resuspended in 100 μL PBS/Matrigel (1:1; Thermo Fisher) and subcutaneously (s.c.) injected in the right flank of the NOD. Cg-Prkdcscid Il2rgtm1Wjl/SzJ (NSG) mice. Once tumors were established (tumor diameter of ≥5 mm), the mice were randomly assigned to either a control group (2 mice receiving sham treatment) or a therapy group (1 mouse per model receiving DSF at 50 mg/kg body weight and trametinib at 0.3 mg/kg body weight). Treatments were applied by oral gavage once daily for 10 days. The control group animals received the vehicle as sham treatment (100 µl vehicle DMSO/ Cremophor®EL (Sigma Aldrich)/ NaCl 0.9% (Braun) in a ratio of 0.5:2.0:7.5 per 20 g of mouse weight). Drinking water was supplemented daily with copper gluconate (1.2 µg/mL) from 7 PM to 7 AM.

To assess melanoma growth in vivo, 1×10^6^ TÜMEL173 melanoma cells in 100 μL PBS/Matrigel (1:1; Thermo Fisher) were s.c. injected into NSG mice as described above. Mice were randomly assigned into four groups (6 in the control group, 5 in the DSF (50 mg/kg body weight) group, 5 in the trametinib (0.3 mg/kg body weight) group, and 6 in the DSF (50 mg/kg body weight) plus trametinib (0.3 mg/kg body weight) group). Therapies were administered by oral gavage once a day (q.d.) for a maximum of 35 days. The control group received the appropriate volume of vehicle (100 µl vehicle DMSO/ Cremophor®EL (Sigma Aldrich)/ NaCl 0.9% (Braun) in a ratio of 0.5:2.0:7.5 per 20 g of mouse weight) as sham treatment. The drinking water of all the groups of mice was supplied with additional copper gluconate (1.2 µg/mL) from 7 AM to 7 PM.

Tumor growth was monitored three times a week (t.i.w.) by caliper measurements. Tumor volumes were calculated with the following formula: volume = (length × width × width/2).

### ^64^CuCl_2_ positron emission tomography (PET) and anatomical magnetic resonance imaging (MRI)

^64^CuCl_2_ (5.75±0.19 MBq) was injected into the tail vein (i.v.) of the respective mice. PET and MR images were acquired 30 min and 6 hours later using a small animal PET scanner (Inveon, Siemens Preclinical Solutions) and a 7 T small animal MR tomograph (Bruker Biospin MRI). PET images were fused to the respective MR images and analyzed by using Inveon Research Workplace software (Siemens Preclinical Solutions). To determine the specific ^64^Cu^2+^ uptake into the tumors, an ex vivo biodistribution of the tumor tissue was performed by γ-counting (Wizard Gamma Counter, Perkin-Elmer). Blood was counted as a control for free ^64^CuCl_2_ in the circulation.

### RNAseq experiment

Total RNA from melanoma cells was isolated using the total RNA Kit (Machery & Nagel) and used for paired-end RNA-seq. Quality was assessed with an Agilent 2100 Bioanalyzer (RIN > 9.5). For library preparation, polyA capture from 100 ng of total RNA using the NEBNext Poly(A) mRNA Magnetic Isolation Module (NEB) was done. Libraries were prepared using the Ultra II Directional RNA Library Prep Kit for Illumina according to the manufacturer’s instructions. The libraries were denatured, diluted to 270 pM and sequenced as paired-end 100 bp reads on an Illumina NovaSeq 6000 platform at a depth of approximately 25 mio reads each. The read quality of RNA-seq data in fastq files was assessed using FastQC (v0.11.4). Reads were aligned using STAR (v2.7.0a), allowing gapped alignments to account for splicing against the Ensembl H. sapiens genome v95. Alignment quality was analyzed using samtools (v1.1). Normalized read counts for all genes were obtained using BEAVR [52]. Transcripts covered with more than 10 reads in at least one group were analyzed to determine differential expression. We set |log2 fold-change| ≥ 0.5 and FDR ≤ 0.05 to call differentially expressed genes. Gene-level abundances were derived as normalized count per million and used for calculating the log2-transformed expression changes underlying the expression heatmaps for which ratios were computed against mean expression in control samples.

### Statistical analysis

GraphPad Prism version 9.4.1. (GraphPad Software, Boston, MA) was used for the statistical analysis of the data. Unless otherwise stated, data are presented as the means with the appropriate standard error of the mean (SEM) or standard deviation (SD). The statistical tests applied are mentioned in the corresponding figure legends. *P*-values <0.05 were considered statistically significant and were labeled as follows: * for *p* < 0.05, ** for *p* < 0.01, *** for *p* < 0.001 and **** for *p* < 0.0001.

*Further methods* can be found in the Supplementary file, especially if they refer only to the Supplementary figures.

## Results

### The MEK inhibitor trametinib inhibits proliferation but is inefficient in apoptosis induction in BRAF WT melanoma cells

**Fig. 1:**
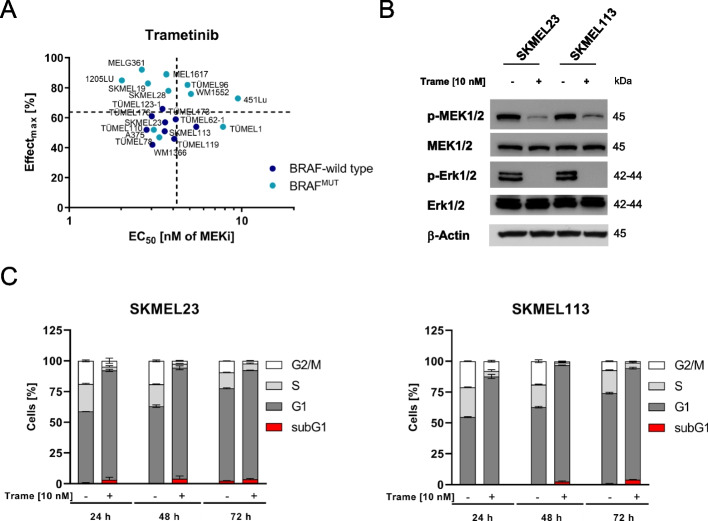
Trametinib inhibits MEK1/2 without marked cytotoxicity or apoptosis induction in vitro. **A** Maximum observed viability reduction (Effect_max_ in %; 100 % corresponds to a complete loss of cell culture viability) and concentration at which half-maximal effective inhibition is observed (EC_50_ in nM) in BRAF^mut^ and BRAF WT melanoma cells were calculated from cell viability data (MUH assay) after 72 hours of treatment with increasing concentrations of trametinib (up to 2 µM). Signals were normalized to the untreated controls. Trametinib EC_50_ values were calculated in GraphPad Prism. Dashed lines represent the mean values of all data points. **B** Cropped immunoblots of MAPK pathway activity (phospho-ERK1/2^Thr202/Tyr204^ and phospho-MEK1/2^Ser217/221^) in the BRAF WT melanoma cell lines SKMEL23 and SKMEL113 after treatment with trametinib (10 nM) for 6 hours. β-Actin served as the loading control. **C** Flow cytometric cell cycle analysis of BRAF WT melanoma cells following treatment with trametinib. BRAF WT melanoma cells were treated with trametinib (10 nM) for 24, 48 and 72 hours (mean ± SD, *n* = 3)

To test the sensitivity of melanoma cells to the MEK inhibitor trametinib, viability assays were performed using different melanoma cell lines (SKMEL23, SKMEL113 and WM1366) and PDSCs (TÜMEL62.1, TÜMEL110, TÜMEL119, TÜMEL123-1, TÜMEL173 and TÜMEL176). In short-term viability assays, trametinib at nanomolar concentrations reduced melanoma cell viability in BRAF WT melanoma cell models by approximately 50% (mean maximum effect 54% [46-63%]), whereas in melanoma cells with BRAF mutations, significantly higher reduction rates (*p*=0.011, Mann-Whitney test) up to 89% (mean maximum effect 64%, [47-89%]) were observed. The average EC50 values did not differ between BRAF WT (3.6±0.7 nM) and BRAF mutated (4.6±2.7 nM; *p*=0.82, Mann-Whitney test) melanoma cells (Fig. 1A and Supplementary Fig. 1). Microscopically, trametinib-treated cells survived the three days of treatment but did not proliferate.

To evaluate the efficacy of trametinib in BRAF WT melanoma cells on a molecular level, MAPK signaling activity was assessed after treatment with the MEK inhibitor. Thr202/Tyr204 phosphorylation of ERK1/2 was efficiently reduced after 6 hours of treatment in the SKMEL23 and SKMEL113 cell lines. Likewise, decreased MEK1/2 phosphorylation at Ser217/221 was observed after treatment with trametinib (Fig. 1B). Although complete inhibition of the MAPK pathway was already achieved at nanomolar concentrations of trametinib, there was almost no induction of apoptosis in BRAF-WT melanoma cells within three days of treatment with MEKi, as indicated by the absence of sub-G1 cells in the cell cycle analysis (Fig. 1C).

### CuET increases the sensitivity of BRAF WT melanoma cells to the MEKi trametinib and enhances its antitumor activity

**Fig. 2:**
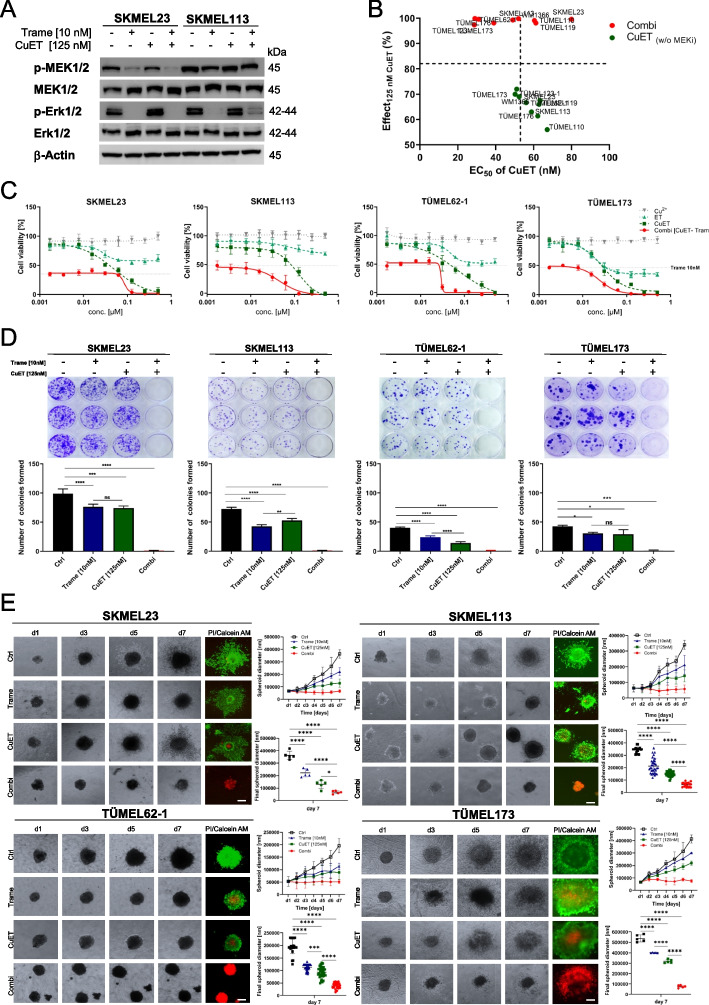
Trametinib plus CuET reduces melanoma cell viability in 2D and 3D BRAF WT melanoma cell models. **A** Cropped Immunoblots of MAPK pathway activity (phospho-ERK1/2^Thr202/Tyr204^ and phospho-MEK1/2^Ser217/221^) in the BRAF WT melanoma cell lines SKMEL23 and SKMEL113 after treatment with trametinib (10 nM), CuET (125 nM) or their combination for 6 hours. β-Actin served as the loading control. **B** Percent viability reduction values (y-axis: Effect_125 nM CuET_) in response to 125 nM CuET (green symbols) and its combination with 10 nM trametinib (red symbols) in BRAF WT melanoma cells are shown together with corresponding EC_50_ values (x-axis). Dashed lines represent the mean values of all data points. Data were calculated from cell viability assays (MUH assay) of BRAF WT melanoma cells after 72 hours of treatment. Effect values (100 % corresponds to a complete loss of cell culture viability) and EC_50_ values (nM) were determined with a dose-response curve using GraphPad Prism. (*n* = 3 independent experiments; mean values). **C** Cell viability assays of four BRAF WT models that were treated with increasing concentrations of Cu^2+^ (up to 500 nM), ET (up to 500 nM) and CuET (up to 500 nM) alone or in combination with 10 nM trametinib for 72 hours. Viability measurements were normalized to the untreated control (*n* = 3 independent experiments measured in hexaplicates; mean ± SD). Dotted lines represent the effect of 10 nM trametinib on cellular viability. **D** Clonogenic growth assay of BRAF WT melanoma cells after 14 d of treatment with trametinib (10 nM), CuET (125 nM) or their combination. Representative results of crystal violet staining are shown. Three independent experiments were quantified, and differences between the treatment groups were analyzed by two-way ANOVA with subsequent Tukey’s multiple comparisons test. **E** Invasive growth of 3D spheroids generated from BRAF WT melanoma cells (SKMEL23, SKMEL113, TÜMEL62-1 and TÜMEL173). Spheroids were embedded into a type I collagen matrix and treated with 10 nM trametinib, 125 nM CuET or their combination for 7 days. Light microscopic images were taken from uniform spheroids every day from day 1 to day 7. Diameter, size and invasion into the collagen of the spheroids were measured with ImageJ by evaluating the diameter and area of the spheroids and normalizing it to the initial spheroid at day 1 (mean ± SD, *n* = 5 for SKMEL23 and TÜMEL173, n≥11 for SKMEL113, n≥23 for TÜMEL62-1, per group). Calcein-AM/PI staining was performed on day 7, and immunofluorescence microphotographs of representative spheroids were taken to determine live or dead cells. White scale bars represent 100 µm. The results were analyzed using Kruskal-Wallis with Dunn’s multiple comparisons test

Next, we investigated the effects of CuET as the major metabolite of DSF on trametinib treated BRAF WT melanoma cells. Again, trametinib inhibited MEK1/2 and ERK1/2 phosphorylation in BRAF WT melanoma cells, which remained largely blocked by the combination (Fig. 2A). In the short-term viability assay, CuET showed a dose-dependent killing effect on BRAF WT melanoma cell lines (SKMEL23, SKMEL113, WM1366) and BRAF WT PDSC lines (TÜMEL62-1, TÜMEL110, TÜMEL119, TÜMEL123-1, TÜMEL173, TÜMEL176). The combination of the MEKi trametinib plus 125 nM CuET achieved a remarkable viability reduction of more than 90% (mean effect 99%, [97-100%], mean EC50 47.8±17.6 nM) at concentrations where trametinib or CuET as monotherapy showed inhibition rates of only 50% and less than 72% (mean effect 66%, [56-72%], mean EC50 58.2±6.1 nM), respectively. Taken together, the addition of at least 125 nM CuET to trametinib resulted in a complete inhibition of cell viability in BRAF WT melanoma cells after three days (Fig. 2B and C, Supplementary Fig. 2A). The enhanced cytotoxic effect with massive melanoma cell death by the combination was demonstrated after 24 hours using live cell imaging with CellTox^TM^ Green, as shown in the example of SKMEL113 (Supplementary Movie 1 and 2). Of note, normal skin cells such as keratinocytes, fibroblasts, and melanocytes were inhibited in their growth by MEKi, but showed no further reduction in viability by additional CuET treatment for three days (Supplementary Fig. 2B). Consistent with the super-additive effect in the short-term viability assays, the combination treatment of melanoma cells significantly reduced colony formation and colony growth in a long-term observation over 14 days. This showed that the combination persistently inhibited BRAF WT melanoma cells in two-dimensional (2D) in vitro models (Fig. 2D).

To verify whether trametinib plus CuET affects the survival of BRAF WT melanoma cells in a more physiological three-dimensional (3D) environment, we evaluated the cellular viability of melanoma spheroids in a short-term viability assay. In line with the monolayer cultures, the MEKi trametinib caused approximately 50% viability reduction in the 3D spheroid models, which was significantly enhanced by the addition of CuET. The combination treatments with 10 nM trametinib resulted in complete killing of the spheroids at concentrations of CuET ≥125 nM (Supplementary Fig. 2C).

To further refine the 3D model and evaluate the long-term effects, we embedded BRAF WT melanoma 3D spheroids in a collagen I matrix before treatment and monitored them over time. MEKi only marginally inhibited the invasive growth of the matrix-embedded spheroids, while the combination completely abolished growth and invasion into the collagen matrix (Fig. 2E). To visualize the cytotoxic effects, the spheroids were stained with calcein-AM and propidium iodide (PI) after 7 days of treatment. Both monotherapies showed only marginally reduced viability and modest induction of cell death. In contrast, spheroids treated with the combination lost their vitality and were completely positive for the death stain PI (Fig. 2E). These results highlight the tumor-killing potential of trametinib in combination with CuET against BRAF-WT melanomas even under physiological 3D culture conditions.

To confirm the effects, we further investigated the efficacy using tumor tissue ex vivo. For this purpose, we prepared tumor slice cultures from ten PDX tumors of NSG mice, all derived from BRAF-WT metastases of patients. Slice cultures were treated with the monotherapies and the combination as before. Resazurin viability assays of melanoma slices treated for 72 hours showed a significant reduction in viability after the combination treatment all ten cases when compared with the controls. Furthermore, in 9 of 10 cases, the viability reduction was significantly enhanced due to the combination compared with trametinib alone (Supplementary Fig. 3A). Calcein-AM/PI stainings confirmed the cytotoxic effects previously observed in 2D and 3D BRAF WT melanoma models (Supplementary Fig. 3B).

### Trametinib plus CuET induces the intrinsic apoptosis pathway in BRAF WT melanoma cells

**Fig. 3:**
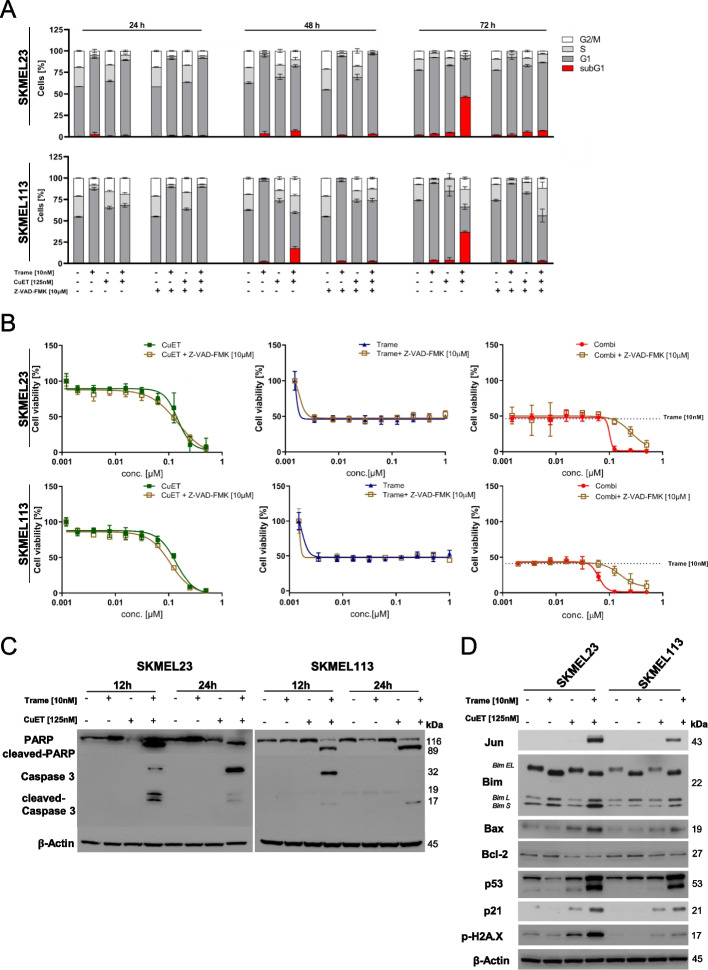
Apoptosis induction by trametinib plus CuET in BRAF WT melanoma cells. **A** BRAF WT melanoma cells were treated with trametinib (10 nM), CuET (125 nM) or their combination in the presence or absence (+/-) of the pan-Caspase inhibitor Z-VAD-FMK (10 µM). Cell cycle analysis at 24, 48 and 72 h was performed, and the cell cycle distribution was plotted with apoptotic cells (sub-G1 fraction) shown as red bars. Three independent experiments were performed (mean ± SD, *n* = 3 independent experiments). **B** Cell viability assays (MUH assay) of BRAF WT melanoma cells (SKMEL23 and SKMEL113) after 72 hours of treatment with increasing concentrations of trametinib (up to 2 µM), CuET (up to 500 nM) or their combination with and without (+/-) the pan-Caspase inhibitor Z-VAD-FMK (10 μM). Signals were normalized to untreated controls (*n* = 3 independent experiments; mean ± SD). Dotted lines represent the effect of 10 nM trametinib on cellular viability. **C** BRAF WT melanoma cells were treated with trametinib (10 nM), CuET (125 nM) or their combination for 12 and 24 hours. Immunoblots (cropped) of whole cell lysates showing cleavage of Caspase-3 and PARP compared to β-Actin. A representative result of two independent experiments is shown.** D** Lysates at 12 hours after treatment were prepared, and immunoblot detections for JUN, BIM isoforms (extra-long: *EL,* long; *L,* short:* S*), BAX, BCL-2, p53, p21 and phosphorylated phospho-H2A.X were performed. β-Actin served as a loading control. A representative result of two independent experiments is shown

We next tested the effect of the combination on apoptosis induction, by analyzing the impact of the treatment on the cell cycle at 24, 48, and 72 hours. Treatment of BRAF WT melanoma cells with 10 nM trametinib induced G1 arrest at 24 hours and discretely induced apoptotic sub-G1 fractions at 72 hours (5-8%). CuET at 125 nM seemed not to influence the cell cycle. The combination of CuET with trametinib resulted in a slight reduction in the population arrested in G1 compared with trametinib, but at the same time in a significant increase in the proportion of sub-G1 cells, especially at 72 hours, whereas the monotherapies did not induce apoptosis-associated DNA fragmentation in a relevant number of cells (Fig. 3A).

DNA fragmentation resulting from apoptosis is mediated by effector caspases. Therefore, we tested whether caspases were involved in the combination-induced DNA fragmentation and cell death induction. Inhibition of caspase activity with the pan-caspase inhibitor Z-VAD-FMK completely mitigated the super-additive cytotoxicity effect and the induction of sub-G1 cells caused by CuET with trametinib in the BRAF WT melanoma cell lines (Fig. 3A and B). However, Z-VAD-FMK did not abrogate the observed G1 arrest and the 50% reduction in viability of melanoma cells mediated by trametinib, again confirming that MEKi did not induce apoptosis. Next, we analyzed different apoptosis-related proteins by Western blotting. Cleavage of caspase-3 and PARP was detectable in response to the combinational treatment after 12 and 24 hours (Fig. 3C). Trametinib alone did not result in the processing of these proteins. A previous report about the induction of BIM by MEKi [53, 54] led us to investigate whether BIM is upregulated in BRAF WT melanoma cells after treatment with our combination. Trametinib alone increased BIM expression when considering all three isoforms (*EL, L, S*), while CuET at 125 nM showed no effect on any BIM isoform. The combination especially enhanced the level of the cytotoxic, pro-apoptotic S isoform (Fig. 3D). Regarding BAX protein levels, CuET slightly increased BAX expression, whereas MEKi alone had no effect. Similar to BIM S, the combination of trametinib plus CuET further upregulated pro-apoptotic BAX levels. In addition, the combination treatment as well as CuET mono-treatment induced down-regulation of the anti-apoptotic protein BCL2 (Fig. 3D). Furthermore, trametinib plus CuET not only increased the protein levels of the cellular gatekeeper p53 but also of its target p21. Since p53/p21 activation is associated with the DNA damage response, we also analyzed the levels of phosphorylated histone H2A.X (phospho-H2A.X) as a sensitive marker for DNA double-strand breaks (DSBs). We observed a clear upregulation of phospho-ɣH2AX in BRAF WT melanoma cells treated with MEKi trametinib plus CuET, which was higher than with the monotherapies (Fig. 3D). Our data strongly suggest that the cytotoxic effects of trametinib plus CuET were accompanied by an accumulation of DSBs, followed by pro-apoptotic p53 activation in BRAF WT melanoma cells.

### MEK inhibition in combination with CuET upregulates endoplasmic reticulum stress-related genes in BRAF WT melanoma cells

**Fig. 4:**
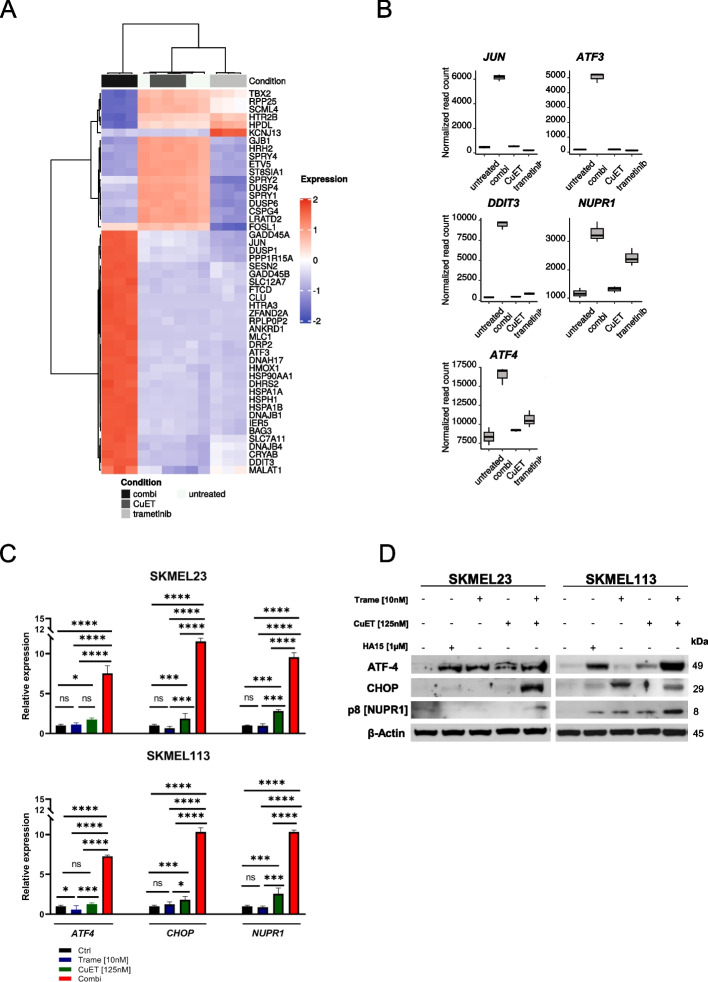
Induction of ER stress-related genes by trametinib plus CuET in BRAF WT melanoma cells. **A** Top 50 differentially expressed genes were clustered in a heatmap showing log10 (fold change expression) values between the monotherapies and the combination using untreated cells as a reference. SKMEL23 cells were treated with trametinib (10 nM), CuET (125 nM) or the combination for 12 hours in triplicate. Total RNA was isolated, and RNA sequencing (RNA-seq) was performed to calculate differentially expressed genes. **B** RNAseq data of SKMEL23 cells revealed the induction of ER stress-related genes due to the combination treatment with trametinib (10 nM) plus CuET (125 nM) for 12 hours (*n* = 3; mean ± SD). **C** Quantitative real-time qPCR using RNA isolated from BRAF WT melanoma cells (SKMEL23 and SKMEL113) treated with trametinib (10 nM), CuET (125 nM) or their combination for 12 hours. The mRNA expression of *ATF4*, *CHOP* and *NUPR1* was normalized to that of TBP and ACTN (*n*=3, mean ± SD). The results were analyzed using one-way ANOVA with subsequent Tukey’s multiple comparisons test. **D** BRAF WT melanoma cells were treated with trametinib (10 nM), 125 CuET (125 nM) and the combination for 12 hours. The ER stress inducer HA15-1 (1 μM) served as a positive control. Western blots (cropped) for ATF4, p8/NUPR1, and CHOP were performed with whole cell lysates, and β-Actin was used as a loading control. A representative result of two independent experiments is shown

To investigate the pathways that could mediate the effects of combined CuET and trametinib treatment, we performed a transcriptome analysis of SKMEL23 cells after 12 hours of treatment. A total of 798 genes were exclusively upregulated (≥4-fold) by the combination of MEKi trametinib plus CuET (Fig. 4A). Among these genes were the ER stress-related genes *ATF3, ATF4, CHOP* and *NUPR1*, the last exhibiting a more than 48-fold increase compared to the control. Similarly, *CHOP* exhibited a 36-fold increase due to the combination treatment. Additionally, *ATF3* and *ATF4* were upregulated >22-fold and >16-fold, respectively, compared to untreated controls) (Fig. 4B). Interestingly, *BIM* also showed significant transcriptional upregulation (>10-fold), which confirmed the previously shown pro-apoptotic mode of action of the combination therapy.

We confirmed the very high upregulation of ER stress-related transcription factor genes such as *ATF4*, *CHOP* and *NUPR1* by combination treatment in the cell lines by real-time qPCR (Fig. 4C). Treatment with trametinib or CuET alone resulted in a partial upregulation of the expression of these genes, but the extent of regulation remained low compared with combination therapy. In line with the RNA data, the combination increased the protein levels of ATF4, CHOP and NUPR1 in BRAF WT melanoma cells even higher than the positive control (GRP78 inhibitor) HA15 (although the detection of NUPR1/p8 was difficult and did not reach a high confidence level). Trametinib did not upregulate the ER stress-related proteins to the same extent with the exception of CHOP in SKMEL23 cells (Fig. 4D).

### Combination of trametinib with CuET induces reactive oxygen species in BRAF WT melanoma cells

ER stress may be the result of disturbed redox homeostasis due to the formation of reactive oxygen species (ROS), and disulfiram and CuET are known to generate ROS [55, 56]. Therefore, we investigated whether ROS were induced in melanoma cells by our treatment and transduced SKMEL23 cells with the biosensor roGFP2-GRX or roGFP2-ORP-1. CuET caused intracellular formation of reactive oxygen species (ROS) in the cells, as indicated by increased biosensor signals. Compared to both monotherapies, the combination of trametinib with CuET significantly enhanced ROS production (Supplementary Fig. 4A and B). Moreover, the addition of the ROS scavenger N-acetyl-cysteine (NAC) abolished the enhancement of cytotoxicity mediated by the combination (Supplementary Fig. 4C and D). Consistent with this, the previous RNA sequencing data showed upregulation of several ROS-associated genes in response to the combination, such as *HMOX1*, *HSPA1*, and *HSPA1B*, also indicating a response to oxidative stress (Fig. 4A).

### JNK/JUN signaling drives apoptosis induction by the combination of trametinib plus CuET

**Fig. 5:**
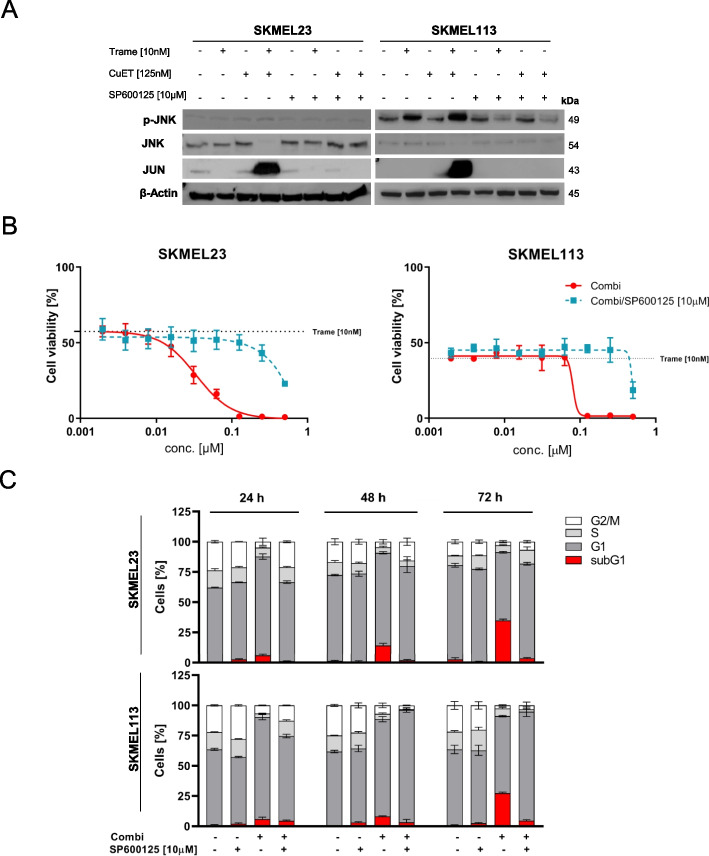
Stress-induced JNK/JUN signaling is crucial for apoptosis induction by the combination of trametinib with CuET. **A** BRAF WT melanoma cells (SKMEL23 and SKMEL113) were treated with the MEK inhibitor trametinib (10 nM), CuET (125 nM), or their combination in the presence or absence (+/-) of the JNK inhibitor SP600125 (10 μM) for 12 hours. Whole cell lysates were used for Western blot analysis of phosphorylated JNK, total JNK, and JUN. β-Actin was used as a loading control. The results shown (cropped) are representative of two independent experiments. **B** Cell viability (MUH assays) of BRAF WT melanoma cells (SKMEL23 and SKMEL113) after treatment for 72 hours with trametinib (10 nM) plus increasing doses of CuET (up to 500 nM) in the presence or absence (+/-) of the JNK inhibitor SP600125 (10 μM). (*n* = 3, mean ± SD). The dotted line indicates the effect of 10 nM trametinib on cellular viability. **C** Flow cytometric cell cycle analyses of BRAF WT melanoma cells (SKMEL23 and SKMEL113) after treatment with MEK inhibitor trametinib (10 nM), CuET (125 nM) and trametinib plus CuET in the presence or absence (+/-) of the JNK inhibitor SP600125 (10 μM) for 24, 48 and 72 hours. Untreated melanoma cells were used as a control. The relative distribution of the cell cycle phases was analyzed (*n* = 3 independent experiments, mean ± SD)

Cellular stressors such as ROS and ER stress activate stress-activated protein kinases (SAPKs), including the JUN N-terminal kinase (JNK) and its target JUN. We therefore investigated whether this JNK/JUN signaling axis was involved in the cell death induced by trametinib plus CuET in melanoma cells. RNA sequencing data initially showed an upregulation of *JUN* gene expression in response to the combination (Fig. 4A). To further investigate whether JNK/JUN signaling was activated by the combination therapy, we analyzed melanoma cells for JNK phosphorylation and JUN accumulation following treatment with trametinib and CuET by Western blotting (Fig. 5A). The combination resulted in a weak increase in Thr183 and Tyr185 phosphorylation of JNK in SKMEL23 and a moderate increase in SKMEL113. JUN protein levels increased significantly in both cell lines. Trametinib or CuET alone did not induce JNK phosphorylation or cause JUN upregulation. The addition of SP600125, a specific JNK inhibitor (JNKi), abolished JNK phosphorylation and also the upregulation of JUN. Thus, the induction of JUN was dependent on JNK activation. Furthermore, JNKi treatment of the cells diminished cytotoxicity and entirely abrogated apoptosis induction due to the combination treatment (Fig. 5B and C). This demonstrates that activation of the JNK/JUN pathway is crucial for combination therapy-mediated apoptosis induction and cytotoxicity in BRAF WT melanoma cells.

We postulated that ER stress and ROS induction were potential triggers for the activation of the JNK/JUN pathway. To explore whether ROS induction mediated by trametinib plus CuET is essential for the upregulation of JUN in BRAF WT melanoma cells, we analyzed JUN expression following treatment with the combination in the presence of the ROS scavenger NAC (Supplementary Fig. 4E). NAC abolished the induction of JUN by combination therapy in both cell lines tested. Additionally, pharmacological chelation of Cu^2+^ with ammonium tetrathiomolybdate (TTM) inhibited ROS induction by the combination and further prevented cytotoxicity and apoptosis induction (Supplementary Fig. 5A and B). Consequently, TTM also prevented the upregulation of JUN (Supplementary Fig. 5C). This indicates that the induction of JUN upon treatment with CuET and trametinib is dependent on Cu^2+^ and ROS, and thus, both factors are essential for combination-induced cytotoxicity and apoptosis in melanoma cells.

### MEK inhibition enhances CuET-induced Cu^2+^ uptake in BRAF WT melanoma cells

To date, diethyldithiocarbamate (ET) and, to an even greater extent its complex with Cu^2+^ have been shown to reduce melanoma cell viability with Cu^2+^ being a key factor in the cytotoxicity . Our results showed that the effects of CuET were completely reversible by the addition of the Cu^2+^ chelators bathocuproindisulfonic acid (BTS) or tetrathiomolybdate (TTM), underscoring the crucial role of Cu^2+^ (Supplementary Fig. 6A and B). However, the effect mediated by trametinib remained unchanged upon the addition of the copper chelators, showing that MEKi acts in a Cu^2+^-independent manner.

**Fig. 6:**
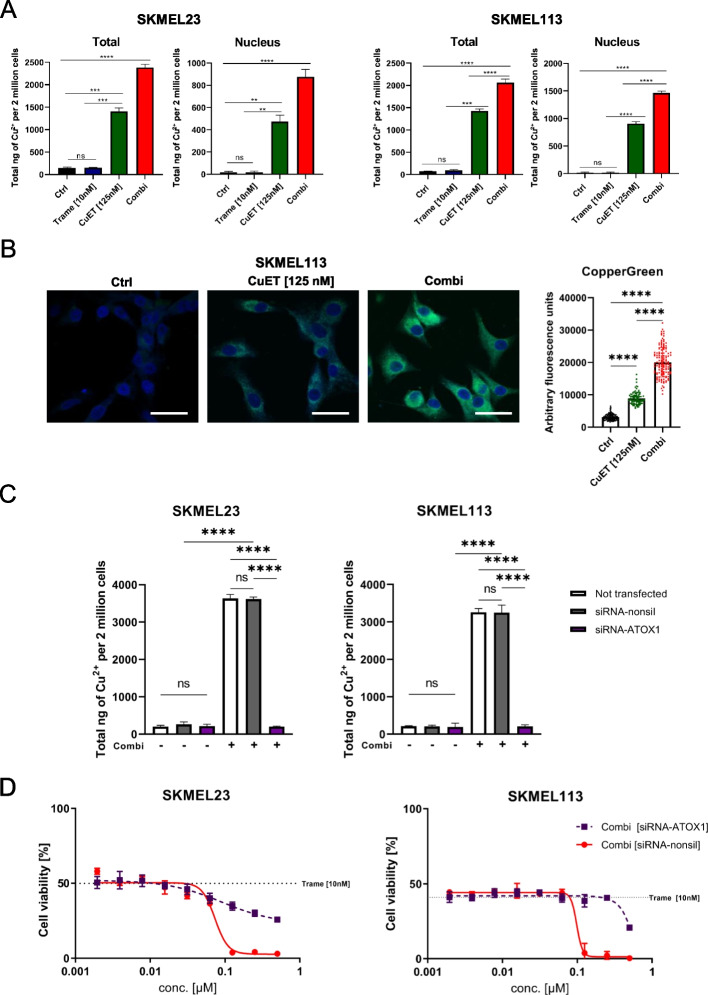
Copper uptake in BRAF WT melanoma cells by CuET and its combination with trametinib. **A** Copper uptake assay measuring Cu^2+^ concentrations in lysates and subcellular subfractions of BRAF WT melanoma cell lines (SKMEL23 and SKMEL113) after treatment with trametinib (10 nM), CuET (125 nM) and the combination for 6 hours. Untreated melanoma cells were used as a control (*n* = 3, mean ± SD). **B** Confocal immunofluorescence analysis for Cu(I) via CooperGreen™ staining in BRAF WT melanoma cells (SKMEL113) after treatment with CuET (125 nM) in the presence or absence of (+/-) 10 nM MEK inhibitor trametinib for 6 hours. Nuclei were stained with DAPI (blue). Scale bars represent 25 μm. Confocal immunofluorescence analysis and fluorescence intensities of the copper probe were recorded as arbitrary fluorescence units (n ≥ 90 cells/ group were analyzed). **C** Spectrophotometric copper uptake assay measuring intracellular Cu^2+^ concentrations in melanoma cell lines (SKMEL23 and SKMEL113) transfected with siRNA directed against ATOX1 or control siRNA followed by treatment with the combination of trametinib (10 nM) plus CuET (125 nM) for 6 hours (*n*=3, means ± SD). **D** Cell viability assay of BRAF WT melanoma cell lines (SKMEL23 and SKMEL113) transfected with control siRNA or siRNA directed against ATOX1 following treatment with the MEK inhibitor trametinib (10 nM) plus increasing concentrations of CuET (up to 500 nM) for 72 hours. Treatment started 24 hours post transfection (*n* = 3; mean ± SD). One-way ANOVA followed by Tukey’s multiple comparisons test was applied in (**A**), (**B**) and (**C**) with * *p* < 0.05; ** *p* < 0.01; *** *p* < 0.001 and **** *p* < 0.0001, ns (not significant)

To investigate whether the treatments directly affected intracellular levels of Cu^2+^ in BRAF WT melanoma cells, we tested whether trametinib, CuET or the combination changed intracellular Cu^2+^ concentrations. CuET and even more pronounced its combination with trametinib significantly increased intracellular Cu^2+^ levels by 6- and 10-fold, respectively (Fig. 6A). To identify the predominant intracellular localization of accumulated copper, subcellular fractionation of melanoma cells following treatment was performed (Supplementary Fig. 5D and E). CuET and the combination with trametinib induced high copper accumulation in the nuclear fraction of the cells, whereas trametinib (and Cu^2+^ alone) neither affected fractionated nor total intracellular copper levels (Fig. 6A). For confirmation, we used the fluorescent dye CopperGREEN^TM^ and performed confocal laser-scanning microscopy (CLSM) on combination-treated SKMEL-113 cells. CuET and, in particular, the combination caused perinuclear Cu^+^ accumulation (note: CopperGREEN^TM^ reacts only with copper (I) not with copper (II)) in BRAF WT melanoma cells after 6 hours of treatment (Fig. 6B and Supplementary Fig. 5E).

### ATOX1 is crucially involved in the uptake of Cu^2+^ by melanoma cells treated with CuET and trametinib

Intracellular copper transport is organized by copper chaperones (ATOX1, CCS, and COX17); however, the transport of copper into the nucleus remains elusive. It has been suggested that ATOX1 may function as a cytoplasmic copper chaperone but also as a transcription factor in the nucleus. We therefore hypothesized that ATOX1 is involved in the combination-induced nuclear copper accumulation in BRAF WT melanoma cells. Downregulation of *ATOX1* via siRNA (Supplementary Fig. 7A and B, Supplementary Table 2) significantly diminished the accumulation of copper in both melanoma cell lines tested after treatment with the combination (Fig. 6C). Thus, the transport of copper into the nucleus was dependent on ATOX1. In parallel to the impaired copper accumulation, knockdown of *ATOX1* significantly reduced the cytotoxic effect of trametinib plus CuET in the BRAF WT melanoma cell lines (Fig. 6D). This suggests that ATOX1 is an important nuclear copper transporter in the cell death mechanism triggered by the combination of trametinib with CuET. Knockdown of the proton-coupled transporters for divalent metal ions *SLC11A1* and *SLC11A2* as known cellular copper transporters did not interfere with the effect of CuET or the combination on SKMEL-23 cells (Supplementary Fig. 7C)

### In vivo effects of the combination in melanoma xenografts

**Fig. 7:**
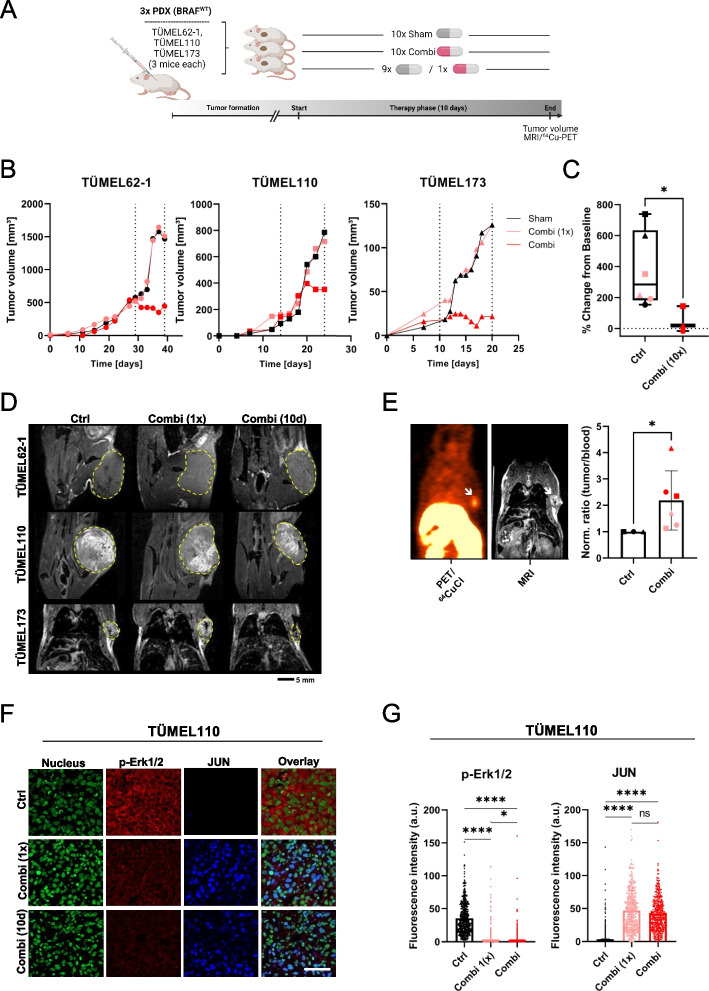
The MEK inhibitor trametinib combined with disulfiram impairs BRAF WT melanoma growth in vivo.** A** Sketch of the NSG mouse experiment with three different patient-derived xenograft (PDX) BRAF WT melanoma models (TÜMEL62-1, TÜMEL110 and TÜMEL173). Three groups were formed: sham treatment; 1x combination treatment (9 days sham and final day combination); and 10x combination treatment (last 10 days combination). DSF (50 mg/kg) plus trametinib (0.3 mg/kg) was orally administered q.d. On the final day, mice received treatment and were subjected to a ^64^Cu-PET/MR scan. **B** Tumor growth curves of PDX BRAF WT melanoma models (TÜMEL62-1 [circles], TÜMEL110 [squares] and TÜMEL173 [triangles]) showed diminished tumor growth under combination therapy of trametinib with DSF (red symbols) versus Ctrl mice (sham group, black symbols; 9x sham and 1x combi light red symbols). The dotted lines indicate the therapy phase (T_x_) of 10 days. **C** Statistical analysis (Mann-Whitney test) of the final tumor volumes for the groups: Ctrl (sham mice and 9x sham plus 1x combi mice; *n* = 6; black and light red symbols); Combi (10x combi; *n* = 3; red symbols). **D** T2-weighted magnetic resonance (MR) imaging was performed on the final day. Representative images for visualization of the combination therapy (1x/10x) or sham-treated subcutaneous TÜMEL110 tumors are shown. Tumors are outlined with yellow dashed lines. **E** Representative PET image of ^64^CuCl_2_ uptake in the tumors (shown is TÜMEL173) of combination-treated mice and corresponding MRI image. Normalized tumor-to-blood ratios (sham treated set as 1) showing the specific accumulation of ^64^CuCl_2_ in combination-treated PDX tumors (TÜMEL62-1 [circles], TÜMEL110 [squares] and TÜMEL173 [triangles]) in vivo 6 h after radiotracer injection. Tumors show an up to 4-fold increased uptake of the radiotracer compared to the blood (Mann-Whitney test). **F** Confocal immunofluorescence analysis of phospho-ERK1/2 and JUN in the tumors. Combination therapy for 10 days diminished ERK1/2 phosphorylation and increased JUN protein levels in the tumors of PDX TÜMEL110 (red color: phospho-ERK1/2; blue color: JUN; green: nuclei /Yopro-1; scale bar 50 µm). **G** Mean fluorescence intensities of phospho-ERK1/2 and JUN staining were used for quantification (≥ 160 cells/group were analyzed). Kruskal-Wallis with subsequent Dunn’s multiple comparisons test. *p< 0.05; ** *p* < 0.01; *** *p* < 0.001; and **** *p* < 0.0001, ns (not significant)

To assess the efficacy of MEKi trametinib plus DSF in vivo, three different BRAF WT PDX models were investigated. NSG mice were subcutaneously injected with the corresponding melanoma cells, and animals were randomized into a control and a therapy group. The control group received sham treatment, and the therapy group received the combination of DSF (50 mg/kg per os, q.d.) and trametinib (0.3 mg/kg per os, q.d.) for ten days with daily tumor measurements to monitor tumor growth (Fig. 7A). Tumor growth was significantly slowed during the first six days after initiation of the combination therapy in the PDX TÜMEL110 and completely abrogated in the PDXs TÜMEL62-1 and TÜMEL173. After 10 days, the therapy group (Combi; *n*=3) showed a significantly reduced tumor growth than the sham treated group (*n*=6) (Fig. 7B and C). MRT scans at day 10 confirmed the decreased tumor volumes due to the therapy (Fig. 7D and Supplementary Fig. 8A). The combination treatment abrogated tumor dynamics in all three PDX models. At day 10, one sham-treated mouse of every BRAF WT model received the oral therapy cocktail (Combi 1x) right before injection of ^64^CuCl_2_ via the tail vain, whereas the other animals received the tail vein injection after their normal treatment regimen (Ctrl and Combi). After the injection of ^64^CuCl_2,_ all mice were transferred to a ^64^Cu-PET/MRT scan. PET imaging showed that copper was also taken up into the tumor (Fig. 7E). Quantitative Ex vivo biodistribution measurements revealed that combination treatment upfront injection of ^64^CuCl_2_ into the tail vein caused an increase in the tumor-to-blood ratio of ^64^Cu^2+^ (Fig. 7E). This confirmed the previously observed cellular copper uptake by melanoma cells after in vitro combination treatment in vivo.

To corroborate the mechanistic results at the cellular and molecular levels, mouse tumors were formalin-fixed and paraffin-embedded for evaluation. Phospho-ERK1/2 stained slides confirmed the the inhibition of the MAPK pathway in all three BRAF WT PDX models (Supplementary Fig. 8C). Additionally, we performed co-staining of phospho-ERK1/2 and JUN to analyze by confocal laser scanning microscopy whether JUN expression was also induced in vivo after 10 days of treatment (Fig. 7F and G, Supplementary Fig. 9A and B). In addition to the observation that all melanomas of mice from the group treated with the combination had reduced phospho-ERK1/2 levels, there was a significant increase in JUN protein levels after the combination treatment with trametinib and DSF in all three BRAF WT PDX models even after the single application of the drugs.

**Fig. 8:**
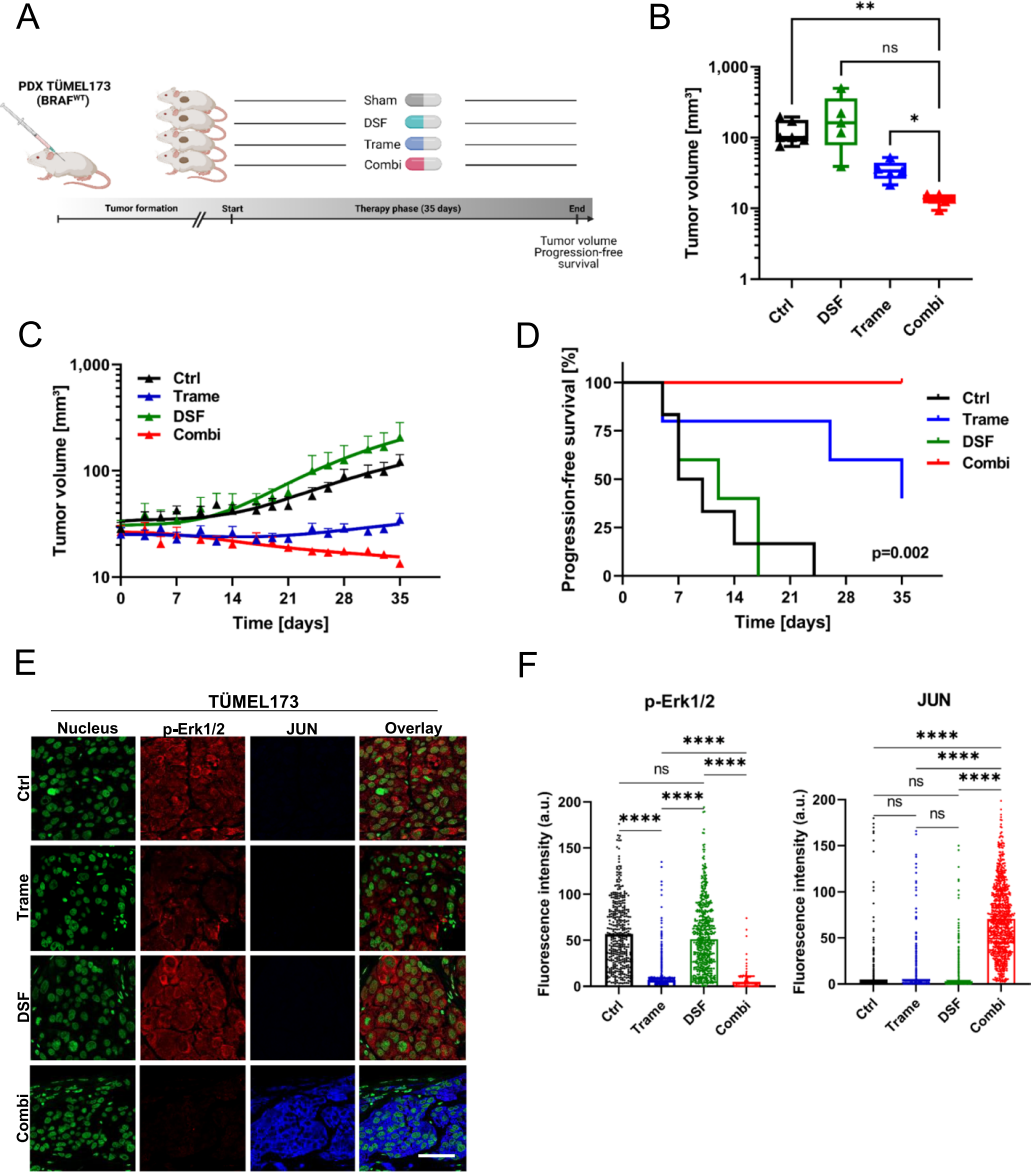
Antitumoral effects of MEK inhibition are significantly enhanced by the combination with disulfiram in a BRAF WT melanoma xenograft model. **A** Sketch of the mouse experiment with four different therapy arms using the BRAF WT PDX melanoma model TÜMEL173. The four different groups were treated daily with vehicle (sham), DSF (50 mg/kg), trametinib (0.3 mg/kg) or their combination for 35 days. **B** Tumor end (day 35) volumes for all the respective therapy groups (control, trametinib, DSF and combination therapy) are shown (control *n*=6, DSF *n*=5, trametinib *n*=5, combination *n*=6). Significance was determined by versions of one-way ANOVA (Welch and Brown-Forsythe) with subsequent Dunnett’s multiple comparisons test. **C** Growth curves of subcutaneous TÜMEL173 melanomas in NSG mice treated with sham (Ctrl) the MEK inhibitor trametinib (trame), DSF and combination (±SEM). **D** Progression-free survival of the animals during the 35 days under treatment. Progression was defined as 20% increase in the tumor volume (log-rank/Mantel-Cox test). **E** Confocal immunofluorescence analysis of phospho-ERK1/2 and JUN in TÜMEL173 tumors after 35 days of treatment with sham (Ctrl), trametinib (Trame), disulfiram (DSF) or the combination (red color: phospho-ERK1/2; blue color: JUN; green: nuclei /Yopro-1; scale bar 50 µm). **F** Fluorescence intensities of phospho-ERK1/2 and JUN staining were used for quantification (≥ 400 cells/group were analyzed). Kruskal-Wallis with subsequent Dunn’s multiple comparisons test. *p< 0.05; ** *p* < 0.01; *** *p* < 0.001; and **** *p* < 0.0001, ns (not significant)

To further investigate the effect of combination therapy on tumor growth, a four-arm mouse experiment was performed comparing the combination of trametinib plus disulfiram with the monotherapies and a sham treatment in the PDX melanoma model TÜMEL173. Treatment was by daily oral administration of the drugs (Fig. 8A). Growth curves showed significant inhibition of tumor growth by trametinib which was further enhanced by the combination therapy. At the end of the experiment, the combination treatment resulted in significantly smaller tumor volumes compared to the other experimental groups (Fig. 8B and C). Within the first two weeks of treatment, we observed a complete stop of tumor growth with the combination, followed by discrete shrinkage of the tumor until the end of the experiment. The latter was not observed in the trametinib group or in the other two groups. Although the growth inhibitory effect of trametinib was significantly greater in vivo than in vitro, tumors in the MEKi group discretely regrew at the end. This is consistent with the development of resistance, which is also observed clinically in patients within a few weeks to months. In contrast, the combination therapy group did not show a reactivation of tumor growth, which can be interpreted as prevention or delay of resistance development to MEK inhibitors due to the combination. In animals treated with DSF alone, tumor growth was not significantly different from that of the control group (Fig. 8B and C). Tumors were histologically confirmed by H&E stained slides (Supplementary Fig. 9C). To analyze whether the difference in tumor growth resulted in altered tumor progression, we calculated progression-free survival based on an increase in tumor diameter of more than 20%, which had to be confirmed on the following day. In the group receiving the combination, there was no disease progression throughout the observation period, and PFS was significantly prolonged compared with the other groups (Fig. 8D). Median PFS was not reached in the combination group, whereas it was 9 days in sham-treated mice, 12 days in DFS-treated mice, and 35 days in trametinib-treated mice. In terms of toxicity, the combination treatment was well tolerated and resulted in normal weight development of the mice (Supplementary Fig. 9D). Similar to the previous experiments, phospho-ERK1/2 and JUN levels were analyzed in TÜMEL173 tumors at 35 days after therapy. Here, only the combination group showed significantly decreased phospho-ERK1/2 levels. An absence in suppression of ERK1/2 phosphorylation in the trametinib-treated tumors (which was detected at day 10 before) suggests reactivation of the MAPK pathway and evolving resistance (Fig. 8E and F). The combination with DSF seemed to prevent BRAF WT melanoma cells from developing secondary resistance to MEKi. JUN protein levels were again significantly increased in tumors of the combination group compared with all other groups. Therefore, the anticancer effect observed in the combination group was again associated with upregulation of JUN (Fig. 8E and F). According to these results, the enhanced tumor control by the combination was confirmed in vivo.

## Discussion

The MAPK kinase pathway is commonly regarded as a central oncogenic signal transduction pathway that is crucial in the development and progression of cutaneous melanomas [57–63]. This is particularly reflected in the frequent mutations in BRAF, NRAS and NF1 [17, 64] but also by activation of the pathway via receptor tyrosine kinases such as epidermal growth factor receptor (EGFR), platelet-derived growth factor receptor (PDGFRA/B), vascular endothelial cell growth factor receptor (VEGFR), hepatocyte growth factor receptor (MET), fibroblast growth factor receptor 2 (FGFR2) or tyrosine-protein kinase KIT/CD117 [64, 65]. In this project, we demonstrated that MEK inhibition was insufficient to induce apoptosis in BRAF WT melanoma cell cultures in vitro. A synergism between the disulfiram metabolite diethyldithiocarbamate in its copper-bound form (CuET) and MEKi was identified. The combination of the MEK inhibitor trametinib with CuET proved to be highly efficient in massively enhancing the anticancer effects at nanomolar concentrations by inducing cell death and apoptosis. The apoptosis induction was dependent on caspase activity and was associated with increased phosphorylated histone H2A.X levels and p53 induced p21 expression, although p21 is a potent universal CDK inhibitor. Our results are consistent with previously published data, which showed an antitumor effect of disulfiram or CuET on cancer cells based on the formation of ROS [66–70], for example, through a Fenton reaction catalyzed by metal ions [71–73]. Similarly, CuET treatment also induced ROS in our BRAF WT melanoma models, and ROS quenchers or potent copper chelators abrogated the therapy-enhancing effect of CuET on the MEKi. This suggests that the combined effects are simultaneously due to the copper ions and the formation of ROS, which are also known to be involved in the mode of action of MAPK pathway inhibition [74–76]. Interestingly, copper ions were found to be essentially involved in the activation of MEK1/2 and thus signal transduction from MAP2K to MAPK [77–79]. This suggests an important role of copper ions in the MAPK signaling pathway, and disruption of copper homeostasis may directly affect its activity. Although higher availability of copper as a cofactor for MEK could increase MAPK activity, this was not the case with our combination treatment although in SKMEL113 the MEK inhibition by trametinib seemed slightly impaired by the combination (Fig. 2A). This difference from our observations could be due to different binding partners or a different compartmentalization of copper. Accordingly, we measured a massive intracellular increase in copper ions with a strong accumulation presumably in the nucleus after treatment of melanoma cells with CuET. Since intracellular transport and copper homeostasis are regulated by transporter proteins (copper transporters 1 and 2, CTR 1 and 2; divalent metal transporter 1, DMT1 and amyloid precursor protein, APP) and copper chaperones (antioxidant 1 copper chaperone, ATOX1; copper chaperone superoxide dismutase, CCS; assembly factor for cytochrome *c* oxidase 11/17, COX11/COX17; and suppressor of COX17 mutation 1/2, SCO1/2) [78, 80], it was reasonable to investigate their involvement in cellular copper uptake. ATOX1 was found to be a key factor in intracellular accumulation and transport into the nuclei containing cellular fraction. Consequently, its downregulation prevented the combination effects of CuET and the copper uptake. The role of ATOX1 as a nuclear copper shuttle has only recently been described [81] but was previously unknown in melanoma cells. Although our cellular fractionation protocol for quantitative copper measurement suggests that significant amounts of copper are translocated to the nucleus, we cannot rule out the possibility that copper accumulation also occurs in additional compartments that we have not studied. In melanosomes, for example, copper is provided by ATP7A for tyrosinase activity [82, 83] . Excess copper is removed from the cell by the ATP-driven copper transporters ATP7A and ATP7B, protecting cells from ROS, and defects can lead to copper storage diseases like Menkes disease [84]. This is especially interesting since a transfer of copper from ATOX1 to ATP7A was described [85, 86]. Therefore, organelles with copper transporters and their role in the cytotoxic effect of DSF during MEK inhibition should be investigated in more detail in the future. This is strengthened by the fact that the copper sensor CopperGreen^TM^ detected mainly cytosolic copper in melanoma cells treated with the combination. However, this reflects probably its exclusive detection of Cu^+^, whereas copper bound to nuclear DNA is mainly present as Cu^2+^ [87].

Oxidative stress, which can be a result of copper and ROS, is well known to interact with endoplasmic reticulum stress (ER stress) [88, 89]. Further, the anti-cancer effect of MAPK pathway inhibition can be massively enhanced in melanoma cells by triggering ER stress [51, 90]. Interestingly, in addition to inhibiting the MAPK pathway, our combination treatment also caused a strong induction of specific ER stress-related genes. This is reminiscent of our previous project, in which BRAF inhibitors acted as potent ER stress inducers in NRAS-mutated melanoma cells to increase apoptosis rates after MEK inhibition [54]. Similar results have been obtained in other cancers, such as hepatocellular, lung, pancreatic, renal, and breast cancers, highlighting the potential of ER stress to induce apoptosis [91–96]. The JNK/JUN signaling axis is also a well-known stress response pathway but remains somewhat ambiguous [97]. JNK activation by ER stress is a commonly described mechanism that can result in apoptosis [98–100]. Simultaneously, JNK can also induce pro-apoptotic genes such as *BID*, *BAX*, *BIM, and BAD* via phosphorylation of JUN [101]. A JNK-dependent phosphorylation of JUN was also shown to lead to downregulation of the anti-apoptotic genes *BCL2* or *BCL2L1* (BCL-xl) [101]. While our experiments with the JNK inhibitor SP600125 clearly revealed that JNK activity is required for apoptosis induction by the combination treatment, no conclusion can be drawn on the relevance of JUN in the observed cell death of our studies at this point. Its induction was detected at both the transcript and protein levels. In line with our finding, early studies showed, that overexpression of JUN can trigger apoptosis in fibroblasts [102] and endothelial cells [103]. Adaphostin as well as the proteasome inhibitor bortezomib were shown to induce apoptosis in multiple myeloma cells causing c-Abl cleavage and caspase activation in a JUN dependent manner [104]. Regarding the mechanism of how JUN may mediate apoptosis, Ferraris et al. identified apoptosis-antagonizing transcription factor (AATF) as a nucleus-restricted cofactor of JUN whose expression level and spatial distribution determined the stress-induced activity of JUN and JUN-mediated apoptosis [105].

Thus, the complete signaling axis might be responsible for apoptosis induction by MEK blockade combined with CuET treatment. However, JUN has also been extensively and very often described as a survival mechanism that suppresses cellular stress-mediated apoptosis and promoting tumor cell survival [102, 106, 107]. Survival-promoting functions of JUN, particularly in the context of the AP-1 transcription factor complex, have also been reported in melanoma cells [108, 109].

An important finding of our studies on the combination therapy of trametinib and disulfiram is its effectiveness in vivo. There was no evidence of severe toxicity at the dosages used in the mouse models. The combination-treated mice maintained their weight, with concomitant suppression of tumor growth and a moderate shrinkage of the tumor. Surprisingly, the MEK inhibitor itself proved to be relatively effective in suppressing tumor growth. In line with our *in vitro* data on apoptosis, no tumor shrinkage was observed with MEK inhibition. PET studies showed accumulation of ^64^Cu^2+^ in the treated tumors, and immunohistochemical staining of the tumors revealed decreased ERK1/2 phosphorylation and increased JUN levels in the tumors. Therefore, we assume similar mechanisms in vivo and in vitro.

In summary, we present the first study on the efficacy and mechanisms of the combined use of the MEK inhibitor trametinib with the well-known alcohol abuse drug disulfiram in malignant melanoma. Both act synergistically together by inducing ROS and ER stress as well as inhibiting MAPK signaling. Cumulatively, this leads to massive tumor cell death with apoptosis induction. Our results are additionally interesting in light of a recent paper showing that disulfiram also enhances T-cell antitumor immunity through direct activation of LCK-mediated TCR signaling [110]. Thus, our data may reveal an interesting new treatment strategy for patients with cutaneous BRAF-WT melanoma who do not respond to current standard therapy.

### Supplementary Information

**Additional file 1. **Supplementary Methods, Supplementary Figures, Supplementary Movies, Supplementary Tables.

## Data Availability

The datasets used and/or analyzed during the current study are available from the corresponding author on reasonable request.
