# Supplementary material for: Augmenting MEK inhibitor efficacy in BRAF wild-type melanoma: synergistic effects of disulfiram combination therapy
Source: J Exp Clin Cancer Res. 2024 Jan 23;43:30. doi: 10.1186/s13046-023-02941-5 (PMC10804659; doi:10.1186/s13046-023-02941-5)
Supplement: Supplementary file 1 — Additional file 1. Supplementary Methods, Supplementary Figures, Supplementary Movies, Supplementary Tables. [file 13046_2023_2941_MOESM1_ESM.zip › Meraz-Torres et al 2023_JExpClinCanRes_Suppl_R01.docx]

**Augmenting MEK inhibitor Efficacy in BRAF Wild-Type Melanoma: Synergistic Effects of Disulfiram Combination Therapy**

(Meraz-Torres et al.)

**SUPPLEMENTARY MATERIAL**

**Supplementary Methods,**

**Supplementary Figures,**

**Supplementary Movies,**

**Supplementary Tables.**

**Supplementary Methods**

*Live-cell imaging with CellTox^TM^ Green*

3 × 10^3^ cells per well were seeded in 96 well plates and adhered in the first 24 hours. Next, treatments were added to the wells in quadruplicates together with the dye CellTox^Tm^ Green according to the manufacturer's protocol. Using an Incucyte SX1, phase contrast and green channels were selected and images of every well were taken all over the observation time.

*Viability (biomass) measurement of 3D spheroids*

The viability of melanoma spheroids was measured using the 4-methylumbelliferyl heptanoate (MUH) assay. Spheroids were created by the previously described hanging drop protocol. Two spheroids were seeded per cavity in 96-well white filter plates with a 0.2 µm PVDF membrane (Corning). After 24 hours, spheroids were treated in hexaplicate for 72 hours. After treatment, the spheroids were washed once with PBS and incubated for 1 hour at 37°C with 100 μg/mL MUH. Viability was quantified by fluorescence (λex/em=355 nm/460 nm) with a Tristar fluorescence microplate reader (Berthold Technologies).

*Resazurin cell viability assay of tissue slice cultures*

Tissue slices were prepared with a thickness of 400 µm using a VT1200S vibratome (Leica). The tissue slices were cultured in RPMI 1640 medium (Gibco) with 10% fetal bovine serum (Sigma Aldrich) and 1% penicillin and streptomycin (Gibco) as described previously. The viability of tissue slice cultures was assessed by the resazurin assay, as described previously. Resazurin is a cell permeable redox indicator and its metabolite resofurin is usually used as a surrogate for cell number or cell mass and therefore viability of the cell culture. After treatment for 96 hours, resazurin (final concentration 0.01 mg/ml) was added to each cavity and incubated for 3 hours at 37°C. Fluorescence measurements (λ_ex_ = 540 nm and λ_em_ = 640 nm) were performed using a Tristar microplate reader (Berthold Technologies) and referenced against a blank sample (medium and resazurin). For microscopic fluorescent viability assessment, slices were stained with 8 µg/mL propidium iodide (Merck) and 2 µM calcein-AM (Merck) and analyzed with an Axioplan 200 fluorescence microscope (Zeiss).

*Immunohistochemical staining*

For the immunohistochemical staining of PDX tumors, tissue was fixed in 4% formaldehyde and embedded in paraffin. Sections (3-5 µm) were prepared using a microtome (Leica) and mounted on SuperFrost Plus slides (R. Langenbrick GmbH) before staining with an anti-phospho-ERK1/2 antibody (1:200, CST, #4370). Further detection was performed using the Lab Vision Liquid Fast-Red Substrate System according to the manufacturer (Thermo Scientific) and counterstained with hematoxylin and eosin.

*Reactive oxygen species (ROS) reporter assay*

Lentiviral particles were produced in 293T cells using the lentiviral transfer vectors pEIGW roGFP2-Grx1 (#64990, Addgene) and roGFP2-Orp1 (#64993, Addgene) in combination with pMD2. G and psPAX2. SKMEL23 cells were transduced at a multiplicity of infection (MOI) of 5 in the presence of 8 μg/mL polybrene, as previously described. Relative H_2_O_2_ levels and the redox status of reduced glutathione (GSH/GSSG) were measured in Orp-1/Grx biosensor SKMEL23 cells by flow cytometry via the fluorescence ratio of Pacific Orange 405 nM/Alexa Fluor 488 nM. pEIGW Grx1-roGFP2 and pEIGW roGFP2-Orp1 were a gift from Tobias Dick (Addgene plasmids # 64990 and 64993).

**Supplementary figure legends**

**Supplementary Fig. 1:** **Trametinib partially impairs the viability of BRAF WT melanoma cells in vitro.** **(A)** Cell viability of BRAF WT melanoma cell lines (SKMEL23, SKMEL113, TÜMEL119, TÜMEL123-1, TÜMEL110, TÜMEL62-1, TÜMEL173, TÜMEL176 and WM1366) was measured 72 hours after treatment with increasing concentrations of trametinib (up to 2 µM). Signals were normalized to the untreated controls (n = 3 independent experiments measured in hexaplicates; mean ± SD).

**Supplementary Fig. 2: Significantly reduced viability of BRAF-WT melanoma cells by the combination of trametinib with CuET. (A)** Cell viability assays of BRAF WT melanoma cells (TÜMEL110, TÜMEL119, TÜMEL123-1, TÜMEL176 and WM1366) were measured after 72 hours of treatment with increasing concentrations of Cu^2+^ (up to 500 nM), ET (up to 500 nM) and CuET (up to 500 nM) in the presence or absence of (+/-) 10 nM trametinib (n= 3, mean ± SD). **(B)** Cell viability assay (MUH assay) of human fibroblasts, melanocytes and keratinocytes after 72 hours of treatment with MEKi trametinib (10 nM), increasing doses of Cu^2+^ (up to 500 nM) and increasing doses of CuET (up to 500 nM) in the presence or absence (+/-) of MEKi trametinib (10 nM) (n= 3, mean ± SD). Signals were normalized to the untreated controls. **(C)** Cell viability assay (MUH assay) of BRAF WT melanoma spheroids (SKMEL23, SKMEL113, TÜMEL62-1 and TÜMEL173) after 72 hours of treatment with MEKi trametinib (10 nM), Cu^2+^ (500 nM), increasing doses of ET (up to 500 nM) and CuET (up to 500 nM) in the presence or absence (+/-) of MEKi trametinib (10 nM). Signals were normalized to the untreated controls (n= 3, mean ± SD).

**Supplementary Fig. 3:** **MEKi trametinib combined with CuET reduces the viability of 3D BRAF WT melanoma slice cultures. (A)** Cell viability of BRAF WT melanoma tumor tissue slices (TÜMEL115-1, TÜMEL115-2, TÜMEL123-1, TÜMEL123-2, TÜMEL158, TÜMEL159, TÜMEL162-1, TÜMEL166/20, TÜMEL173 and TÜMEL169-1) was measured after treatment with trametinib (10 nM), CuET (125 nM) and their combination for 72 hours. One-way ANOVA with subsequent Tukey’s multiple comparisons test. *p< 0.05; ** p < 0.01; *** p < 0.001; and **** p < 0.0001, ns (not significant). **(B)** Immunofluorescent Calcein AM/PI staining of BRAF WT tumor tissue slices (TÜMEL123-1, TÜMEL158 and TÜMEL159) after treatment with 10 nM trametinib, 125 nM CuET and their combination for 72 hours.

**Supplementary Fig. 4: Trametinib combined with CuET induces intracellular reactive oxygen species. (A-B)** Flow cytometry measurement of the cellular redox state of glutathione (GSH/GSSG ratio) and H_2_O_2_ levels in BRAF WT melanoma biosensor cells (SKMEL23-ORP1/SKMEL23-GRX) following corresponding treatments. SKMEL23 biosensor cells were treated with MEKi trametinib (10 nM), CuET (125 nM) and their combination for 1, 6, 12, 24 and 48 hours. Three independent experiments were performed, and representative data are shown (mean ± SD, n = 3). **(C)** Cell viability of BRAF WT melanoma cells (SKMEL23 and SKMEL113) was measured after 72 hours of treatment with MEK inhibitor trametinib (10 nM) in combination with increasing doses of CuET (up to 500 nM) in the presence or absence (+/-) of the potent ROS scavenger N-acetyl-cysteine (NAC; 1 mM) (n = 3 independent experiments, ± SD). Viability was normalized to untreated control cells. **(D)** Cell cycle analyses of BRAF WT melanoma cells (SKMEL23 and SKMEL113) treated with MEKi trametinib (10 nM), CuET (125 nM) or their combination in the presence or absence (+/-) of NAC (1 mM) for 24, 48 and 72 hours. Apoptotic cells (sub-G1 fraction) are shown as red bars. Three independent experiments were performed (n = 3, mean ± SD). **(E)** Western blot of lysates from the SKMEL23 and SKMEL113 cell lines treated with the combination (125 nM CuET plus 10 nM trametinib) for 12 hours in the presence or absence (+/-) of NAC to detect JUN protein levels (cropped pictures).

**Supplementary Fig. 5: Copper plays a critical role in the cellular cytotoxicity and apoptosis induction mediated by combination therapy of trametinib plus CuET. (A)** Cell viability of BRAF WT melanoma cells (SKMEL23 and SKMEL113) was measured after treatment with MEKi trametinib (10 nM) in combination with increasing doses of CuET (up to 500 nM) in the presence or absence (+/-) of the intracellular Cu chelator TTM (500 nM) for 72 hours (n= 3, mean ± SD). Signals were normalized to the untreated controls. **(B)** BRAF WT melanoma cells (SKMEL23 and SKMEL113) were treated with MEKi trametinib (10 nM), CuET (125 nM) or their combination in the presence or absence (+/-) of the Cu^2+^ chelator TTM (500 nM) for 24, 48 and 72 hours. Cell cycle analyses were performed, and the cell cycle distribution was calculated with apoptotic cells (sub-G1 fraction) shown as red bars. Three independent experiments were performed (n = 3, mean ± SD). Untreated melanoma cells were used as a control. **(C)** Western blot of lysates from the SKMEL23 and SKMEL113 cell lines treated with the combination (125 nM CuET plus 10 nM trametinib) for 12 hours in the presence or absence (+/-) of TTM (500 nM) to detect JUN protein levels. **(D)** Western blot analysis of lamin-B1 and β-tubulin in whole-cell protein lysates of BRAF WT melanoma cells (SKMEL113) after the subcellular fractionation process. **(E)** Copper uptake assay measuring Cu^2+^ concentrations in lysates and subcellular fractions of BRAF WT melanoma cell lines (SKMEL23 and SKMEL113) after treatment with Cu^2+^ (250 nM), trametinib (10 nM), ET (125 nM), CuET (125 nM) and the combination of trametinib (10 nM) plus CuET (125 nM) for 6 hours. Untreated melanoma cells were used as a control. Values are means ± SD of three independent experiments (n = 3, mean ± SD). Untreated melanoma cells were used as a control. Significance was determined by one-way ANOVA with subsequent Tukey’s multiple comparisons test. *p< 0.05; ** p< 0.01; *** p < 0.001; and **** p < 0.0001, ns (not significant).

**Supplementary Fig. 6: Cytotoxicity of the combination depends on the availability of copper ions. (A)** Cell viability (MUH assay) of BRAF WT melanoma cell lines (SKMEL23 and SKMEL113) after treatment with increasing doses of ET (up to 500 nM) and CuET (up to 500 nM) in the presence or absence (+/-) of the Cu chelator bathocuproindisulfonic acid BTS (50 µM) for 72 hours. (n = 3; mean ± SD). Viability was normalized to untreated control cells. **(B)** Clonogenic growth assay of the BRAF WT melanoma cell line SKMEL113 after 14 d of treatment with CuET (60 nM, 125 nM and 250 nM) in the presence or absence (+/-) of the Cu chelator TTM (500 nM). Representative results of crystal violet staining are shown. Colonies of three independent experiments were quantified, and differences between the treatment groups were analyzed by two-way ANOVA with subsequent Tukey’s multiple comparisons test. *p< 0.05; ** p< 0.01; *** p < 0.001; and **** p < 0.0001, ns (not significant).

**Supplementary Fig. 7: ATOX1 plays a main role in the cellular cytotoxicity mediated by the combination of trametinib plus CuET. (A)** Western blot analysis for ATOX1 with protein lysates of BRAF WT melanoma cells (SKMEL23 and SKMEL113) prepared at 48 hours post transfection with siRNA directed against ATOX1 or with control siRNAs specific for SLC11A1, SLC11A2, SOX10 or a non-silencing sequence. β-Actin served as the loading control. **(B)** Quantitative real-time qPCR using RNA isolated from BRAF WT melanoma cells (SKMEL23 and SKMEL113) transfected with control siRNA or siRNA directly against ATOX1 at 48 hours after transfection. mRNA expression of ATOX1 was normalized to TBP (mean ± SD of triplicates of three independent experiments). **(C)** Cell viability assays of SKMEL23 cells that were transfected with control siRNA or siRNA directly against ATOX1, SLC11A1 or SLC11A2 48 hours before treatment with increasing concentrations of CuET (up to 500 nM) for 72 hours. (n = 3; mean ± SD). Viability was normalized to untreated transfected cells. **(D)** Cell viability assays of SKMEL23 cells that were transfected with control siRNA or siRNA directly against ATOX1, SLC11A1 or SLC11A2 48 hours before treatment with increasing concentrations of CuET (up to 500 nM) combined with trametinib (10 nM) for 72 hours. (n = 3; mean ± SD). Viability was normalized to untreated transfected cells.

**Supplementary Fig. 8: Analysis of PDX melanomas after 10 days of therapy with combined trametinib and disulfiram. (A)** Volume calculations of the subcutaneous PDX BRAF WT melanomas (TÜMEL62-1, TÜMEL110 and TÜMEL173) from the MRI data (two scans each) after treatment with disulfiram (50 mg/kg) plus trametinib (0.3 mg/kg) for 10 days (Ctrl: sham treatment with vehicle; Combi 1x: one therapy at day 10; Combi 10d: daily therapy for 10 days) **(B)** H&E staining of the subcutaneous PDX BRAF WT melanomas (TÜMEL62-1, TÜMEL110 and TÜMEL173) after treatment with sham or disulfiram (50 mg/kg) plus trametinib (0.3 mg/kg) once on the last day (Combi 1X) or daily for 10 days (Combi) at three different magnifications. **(C)** Immunohistochemical analysis of phospho-ERK1/2 in tumors using FastRed substrate and H&E counterstaining.

**Supplementary Fig. 9: The MEK inhibitor trametinib combined with disulfiram impairs BRAF WT melanoma growth *in vivo*. (A&B)** Confocal immunofluorescence analysis of phospho-ERK1/2 and JUN in the PDX models TÜMEL62-1 and TÜMEL173. Daily combination therapy with trametinib (at 0.3 mg/kg per os) plus disulfiram (50 mg/kg per os) for 10 days diminished ERK1/2 phosphorylation and increased JUN protein levels (red color: phospho-ERK1/2; blue color: JUN; green: nuclei /Yopro-1). Fluorescence intensities of phospho-ERK1/2 and JUN staining were used for quantification (≥ 160 cells/group were analyzed). Significance was determined by Kruskal-Wallis with subsequent Dunn’s multiple comparisons test. *p< 0.05; ** p < 0.01; *** p < 0.001; and **** p < 0.0001, ns (not significant). (**C)** Body weight development of NOD SCID gamma mice during the therapy phase for 35 days with trametinib (at 0.3 mg/kg per os), disulfiram (50 mg/kg per os) and their combination. No significant differences were detected in the four groups. (D) H&E staining of subcutaneous PDX TÜMEL173 melanomas after treatment with disulfiram (50 mg/kg per os), trametinib (0.3 mg/kg per os), the combination or sham for 35 days. Only small melanoma cell nests were found in mice receiving combination therapy.

**Supplementary Tables**

**Supplementary Table 1.** *Patient-derived melanoma models*

**Cell line Origin NRAS^mut^ BRAF^mut^**

**TÜMEL1** lymph node met. - BRAF V600E

**TÜMEL61** lymph node met. - BRAF V600E

**TÜMEL62-1** brain metastasis NRAS Q61R -

**TÜMEL78** skin metastasis - BRAF V600E

**TÜMEL96** brain metastasis - BRAF V600E

**TÜMEL110** endolumenal met. A146T -

**TÜMEL115** lymph node met. - BRAF V600E

**TÜMEL119** lymph node met. - BRAF V600E

**TÜMEL123-1** skin metastasis NRAS Q61R -

**TÜMEL123-2** skin metastasis NRAS Q61R -

**TÜMEL173** adrenal gland met. NRAS G12V -

**TÜMEL176** skin metastasis **- -**

**TÜMEL115-1** lymph node met. - BRAF V600E

**TÜMEL115-2** lymph node met. - BRAF V600E

**TÜMEL158** skin metastasis  **- B**RAF V600K

**TÜMEL159** lymph node met. **- -**

**TÜMEL162-1** skin metastasis NRAS Q61K **-**

**TÜMEL166/20** lymph node met. **- -**

**TÜMEL169-1** skin metastasis - BRAF V600K

**Supplementary Table 2.** *Forward and reverse primer sequences*

**Target Direction Sequence (5’ → 3’) Annealing Supplier**

**temperature**

**β-Actin** forward ttgttacaggaagtcccttgcc 55°C biomers.net

reverse atgctatcacctcccctgtgtgtg 55°C biomers.net

**ATF4** forward tggggaaaggggaagaggttgtaa 60°C biomers.net

reverse agtcgggtttgggggctgaag 61°C biomers.net

**ATOX1** forward ttgttacaggaagtcccttgcc 55°C biomers.net

reverse tctggaagccagcgggaggat 61°C biomers.net

**CCS** forward cagaatggaggatgagcagctg 56°C biomers.net

reverse gagcgtgcaatgatgccacagg 62°C biomers.net

**CHOP** forward aaggcactgagcgtatcatgt 51°C biomers.net

reverse tgaagatacacttccttcttgaacac 52°C biomers.net

**COX 17** forward ttgcccggagaccaagaaggc 62°C biomers.net

reverse attatttattcacacagcagaccac 52°C biomers.net

**CTR1** forward ccaggaccaaatggaaccatcc 59°C biomers.net

reverse accacctggatgatgtgcagca 60°C biomers.net

**DMT1** forward agctccaccatgacaggaacct 57°C biomers.net

reverse tggcaatagagcgagtcagaacc 58°C biomers.net

**P8** forward ccattcctacctcgggcctctcatc 63°C biomers.net

reverse tcttggtgcgacctttccggc 62°C biomers.net

**TBP** forward tgcacaggagccaagagtgaa 60°C biomers.net

reverse cacatcacagctccccacca 62°C biomers.net

**Supplementary Table 3.** *siRNA guide and passenger sequences*

**Gene ID Gene Sequence 5`-3` Nucleotides Supplier**

**SLC11A2** *Guide* UAAUAGUGSUGSGSSSGCCCCC 22 nt Riboxx

*Guide* UAGAAAUAGACUUUGGACCCCC 22 nt

*Guide* AGAAAGUCAUUCAUCCCUGCCCCC 24 nt

*Passenger* GGGGGCUUUCUCAUCACUAUUA 22 nt

*Passenger* GGGGGUCCAAAGUCUAUUUCUA 22 nt

*Passenger* GGGGGCAGGGAUGAAUGACUUUCU 24 nt

**SLC31A1** *Guide* AUAUUUAAACUGGCACCCACCCCC 23 nt Riboxx

*Guide* UAUUUAGUCUCAAACCACCCCC 22 nt

*Guide* AUUAUCACAAUCCAAGAGCCCCC 23 nt

*Passenger* GGGGGUGGGUGCCAGUUAAAUAU 23 nt

*Passenger* GGGGGUGGUUUGAGACUAAAUA 22 nt

*Passenger* GGGGGCUCUUGGAUUGUGAUAAU 23 nt

**ATOX1**  *Guide* AUGUCAUACUUAACUCCUCCCCC 23 nt Riboxx

*Guide* AUACUUAACUCCUCCAAGCCCCC 23 nt

*Guide* UGUCAUACUUAACUCCUCCCCC 22 nt

*Passenger* GGGGGAGGAGUUAAGUAUGACAU 23 nt

*Passenger* GGGGGCUUGGAGGAGUUAAGUAU 23 nt

*Passenger* GGGGGAGGAGUUAAGUAUGACA 22 nt

**Sox10** *Guide* CCGUAUGCAGCACAAGAAA-dTdT 23 nt Riboxx

*Passenger* UUUCUUGUGCUGCAUACGG-dTdT 23 nt

**nonsil** *Guide* AAUUCUCCGAACGUGUCAGU-dTdT 23 nt Riboxx

*Passenger* ACGUGACACGUUCGGAGAAUU-dTdT 23 nt
