# Supplementary figures and images for: Augmenting MEK inhibitor efficacy in BRAF wild-type melanoma: synergistic effects of disulfiram combination therapy
Source: J Exp Clin Cancer Res. 2024 Jan 23;43:30. doi: 10.1186/s13046-023-02941-5 (PMC10804659; doi:10.1186/s13046-023-02941-5)

A

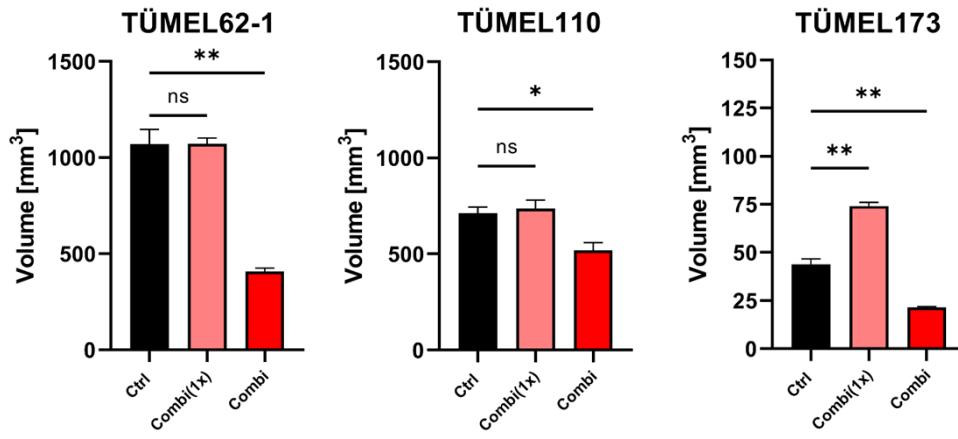

B

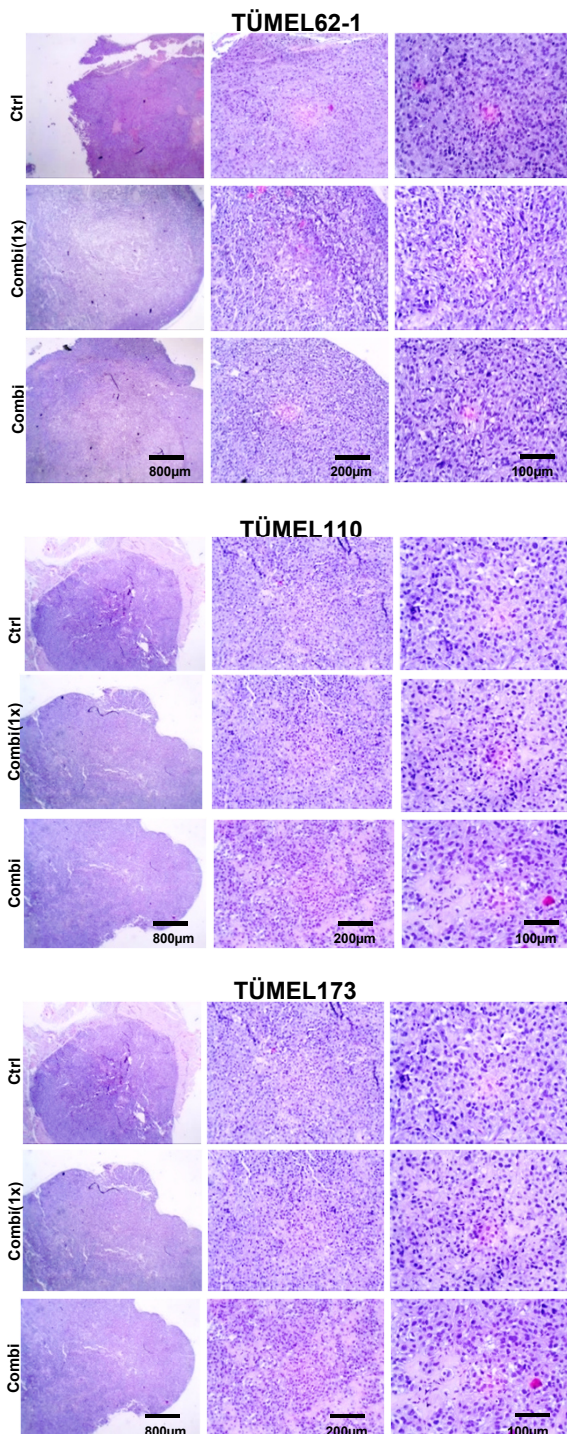

C

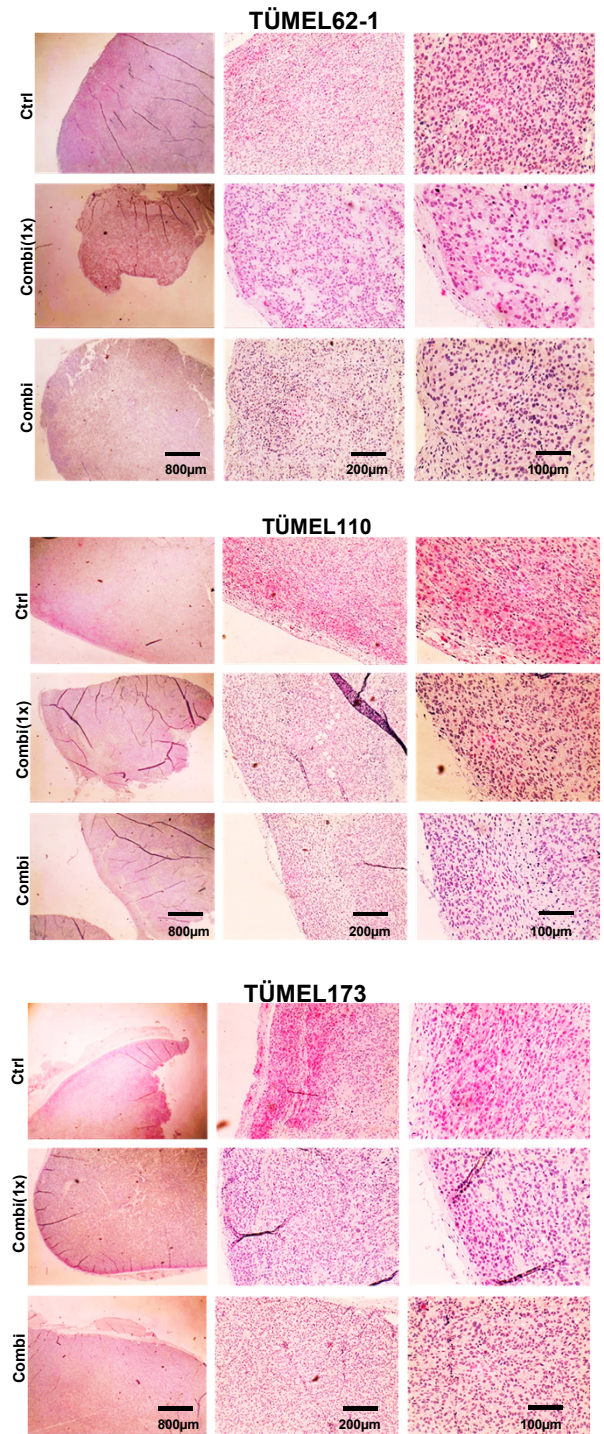

Supplement: Supplementary file 1 — Additional file 1. Supplementary Methods, Supplementary Figures, Supplementary Movies, Supplementary Tables. [file 13046_2023_2941_MOESM1_ESM.zip › Supplemental Figure 8_R2.pdf]

A

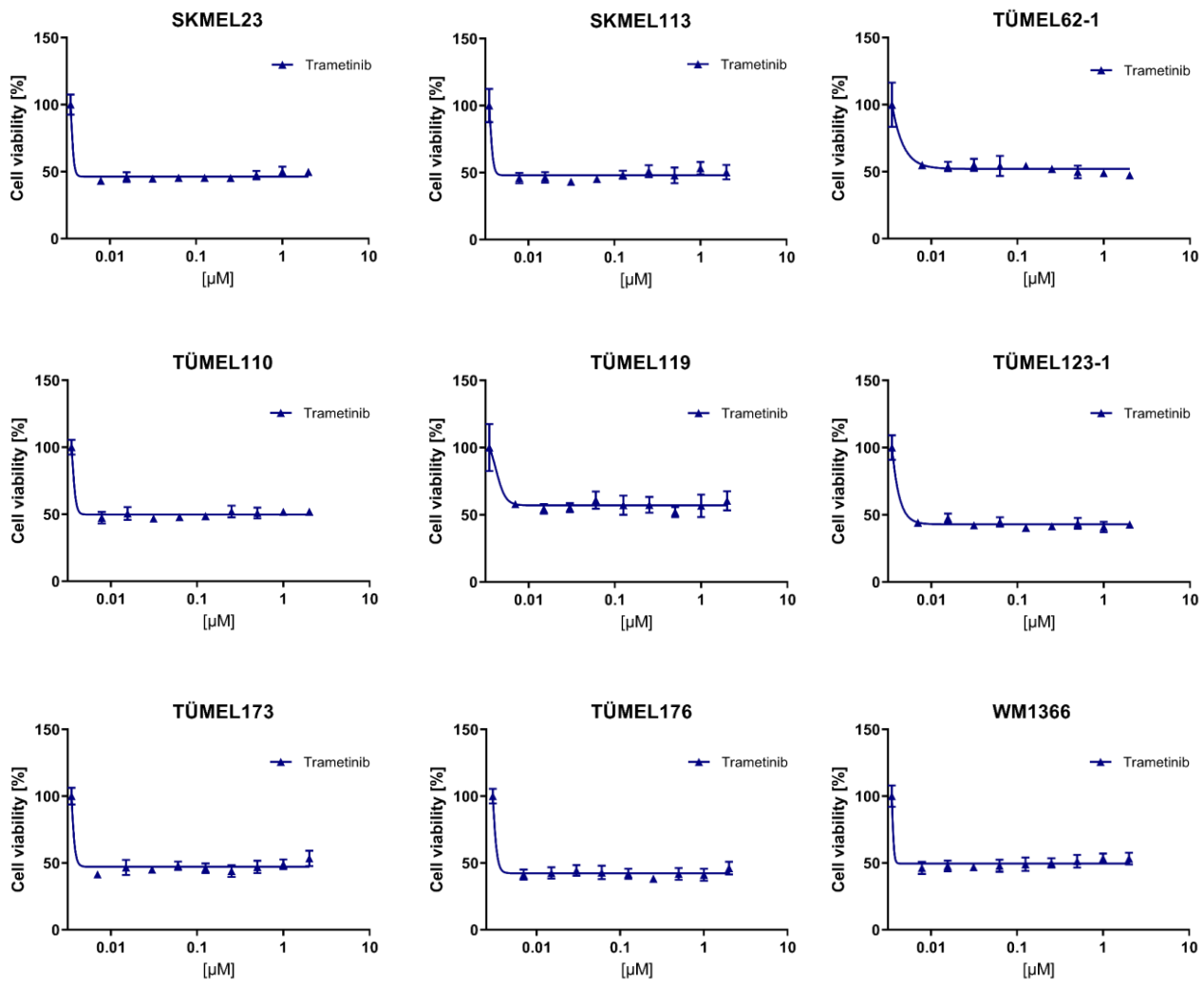

Supplementary Figure 1

Supplement: Supplementary file 1 — Additional file 1. Supplementary Methods, Supplementary Figures, Supplementary Movies, Supplementary Tables. [file 13046_2023_2941_MOESM1_ESM.zip › Supplementary Figure 1.pdf]

**A**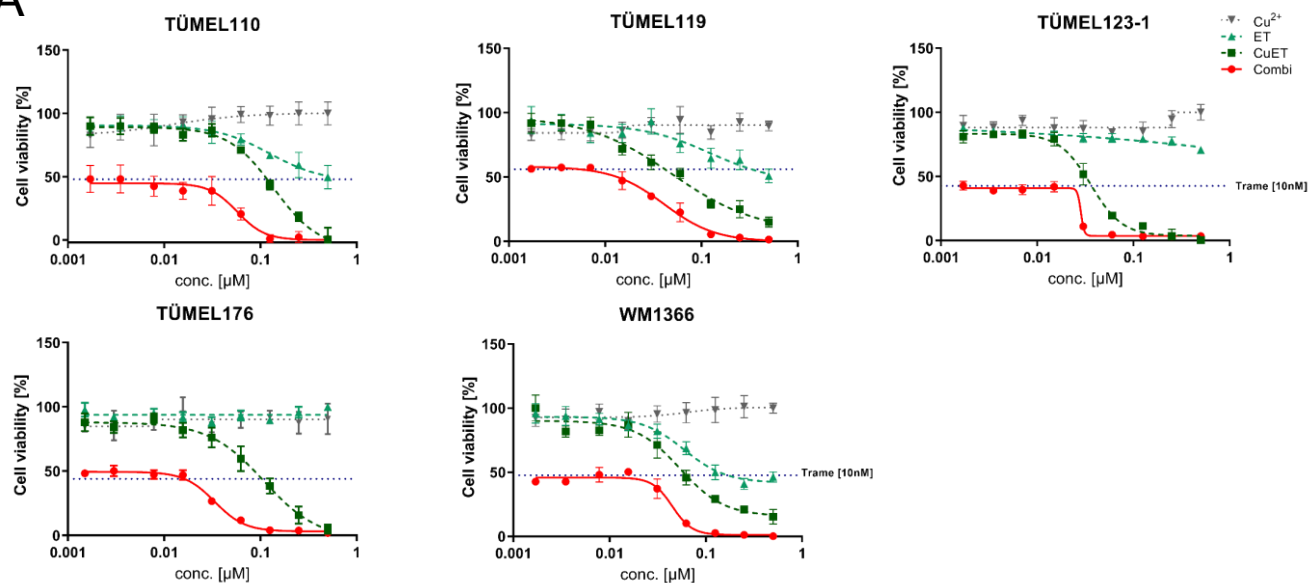**B**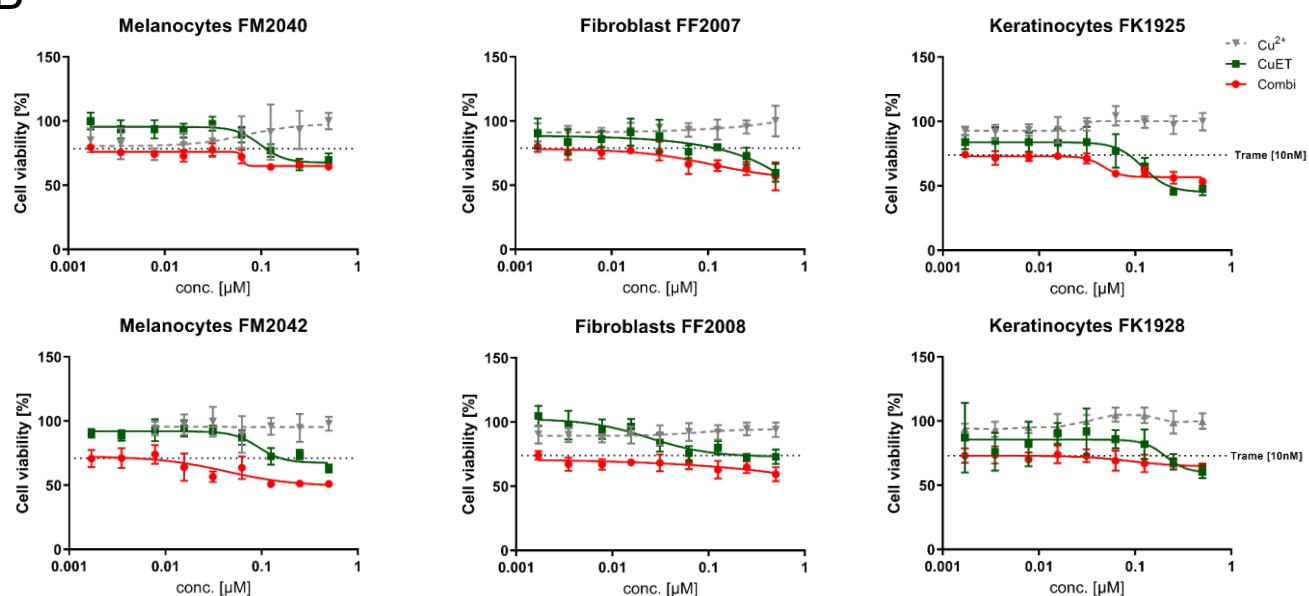**C**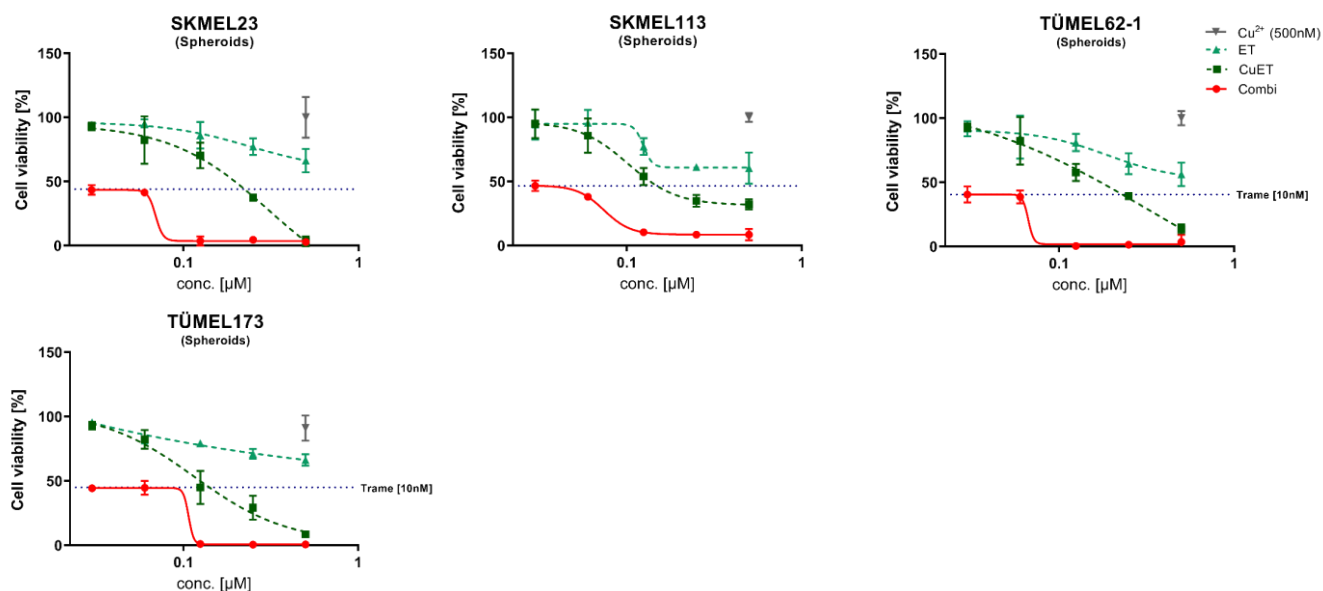

Supplement: Supplementary file 1 — Additional file 1. Supplementary Methods, Supplementary Figures, Supplementary Movies, Supplementary Tables. [file 13046_2023_2941_MOESM1_ESM.zip › Supplementary Figure 2.pdf]

A

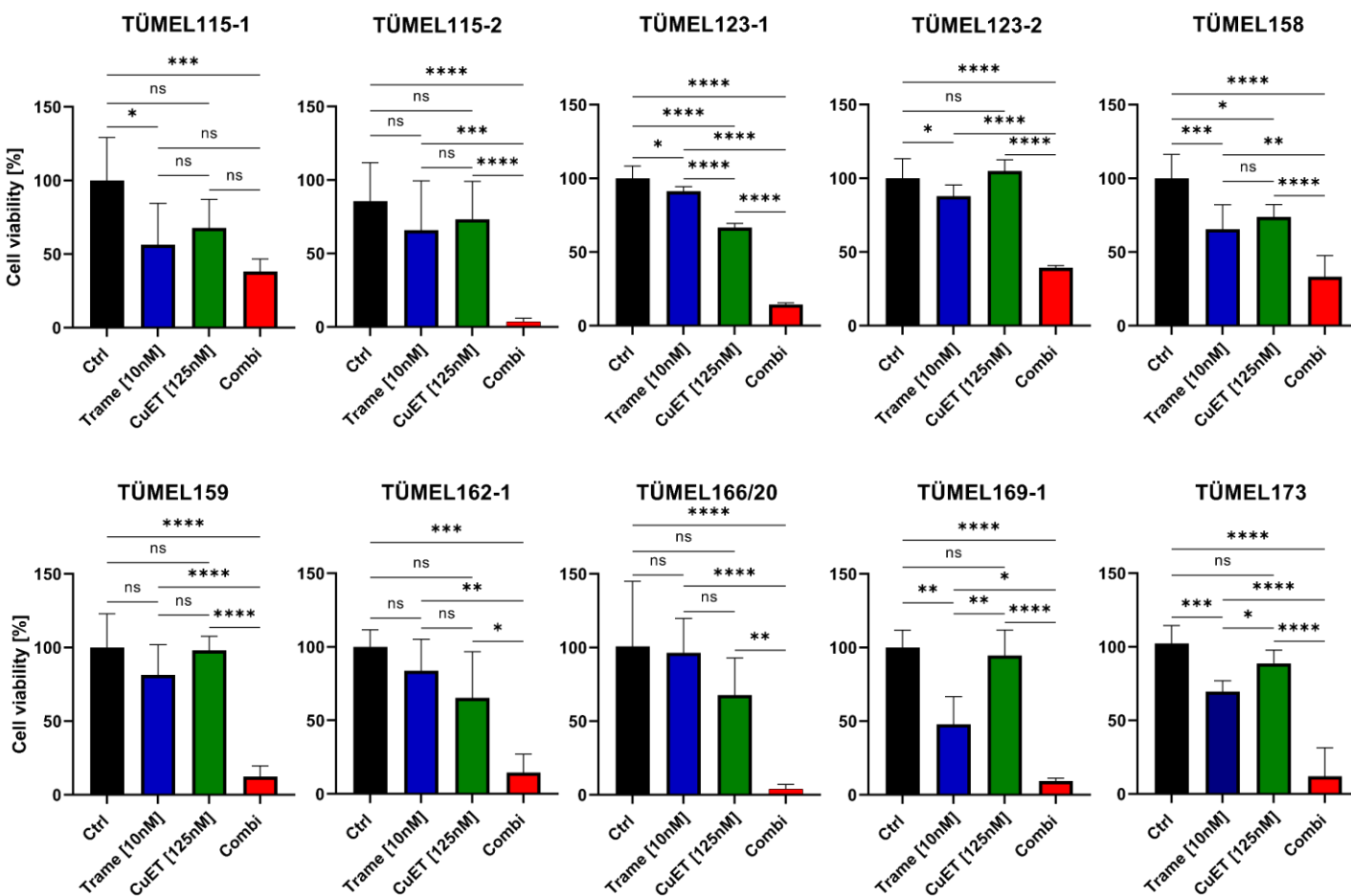

B

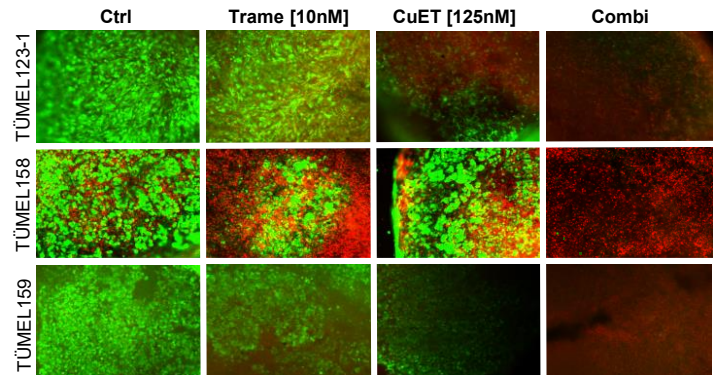

Supplement: Supplementary file 1 — Additional file 1. Supplementary Methods, Supplementary Figures, Supplementary Movies, Supplementary Tables. [file 13046_2023_2941_MOESM1_ESM.zip › Supplementary Figure 3.pdf]

**A**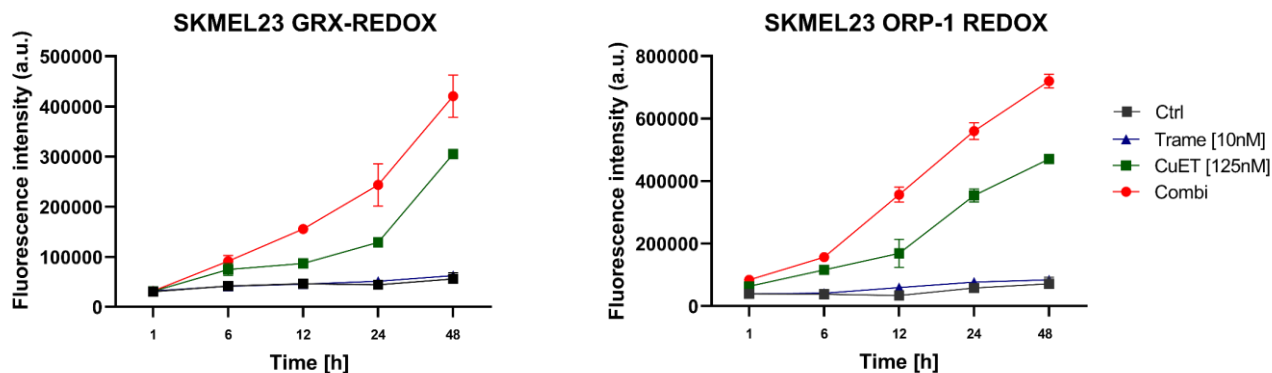**B**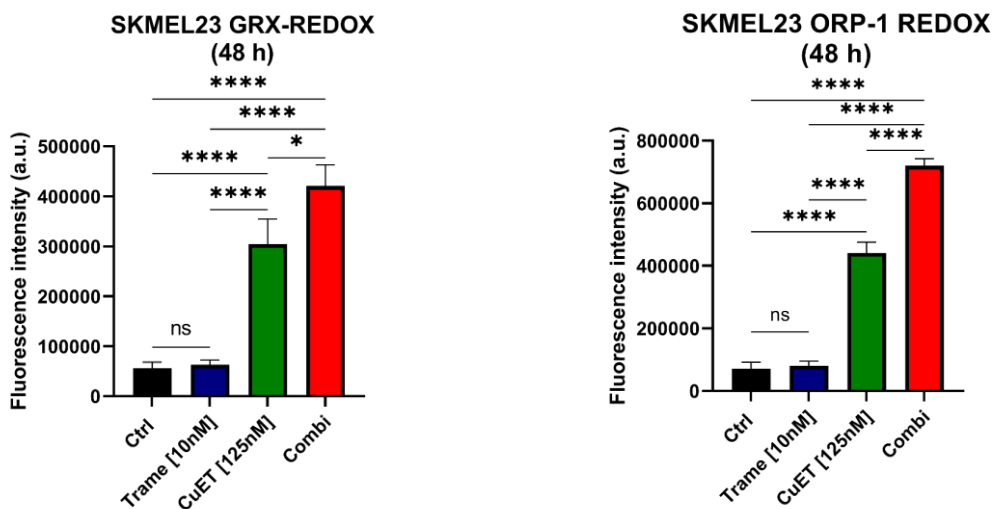**C**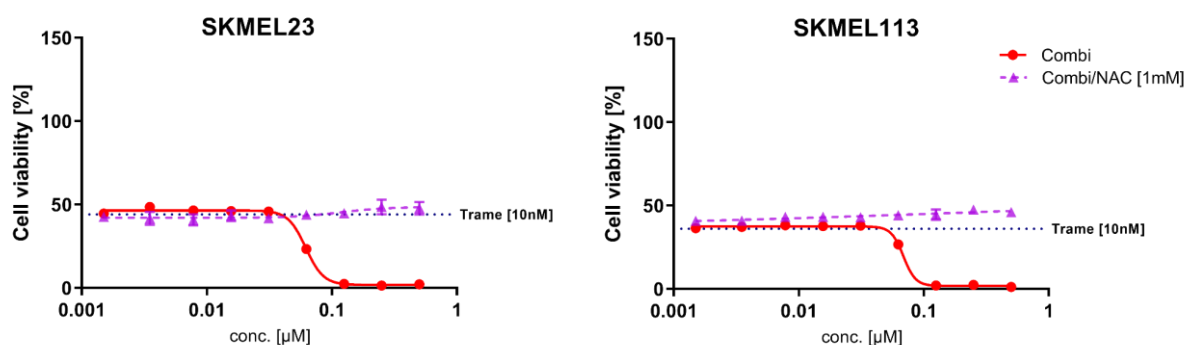**D**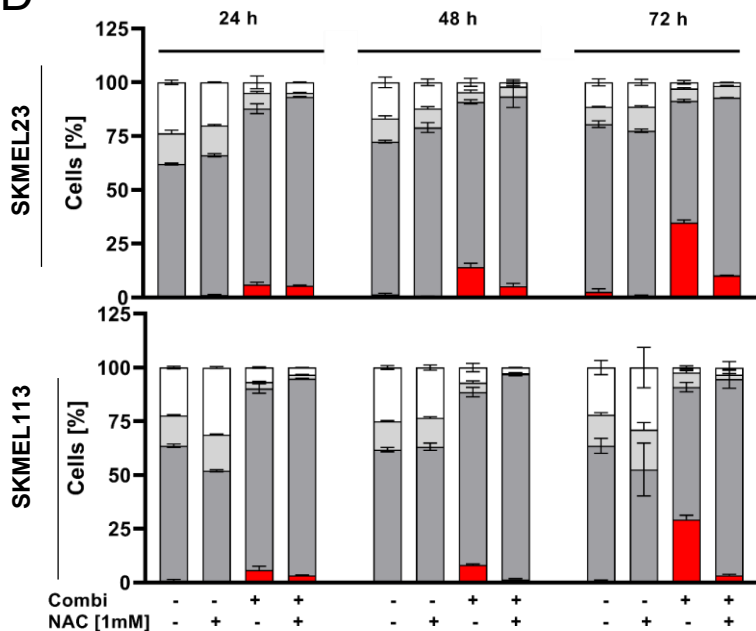**E**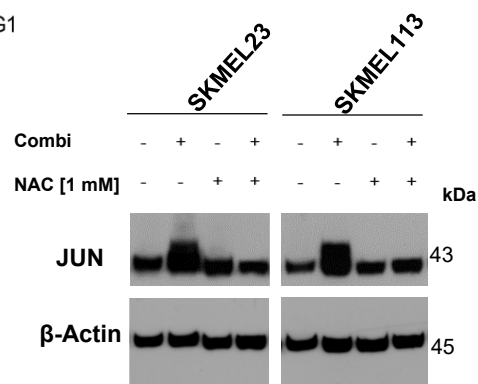

Supplementary Figure 4

Supplement: Supplementary file 1 — Additional file 1. Supplementary Methods, Supplementary Figures, Supplementary Movies, Supplementary Tables. [file 13046_2023_2941_MOESM1_ESM.zip › Supplementary Figure 4.pdf]

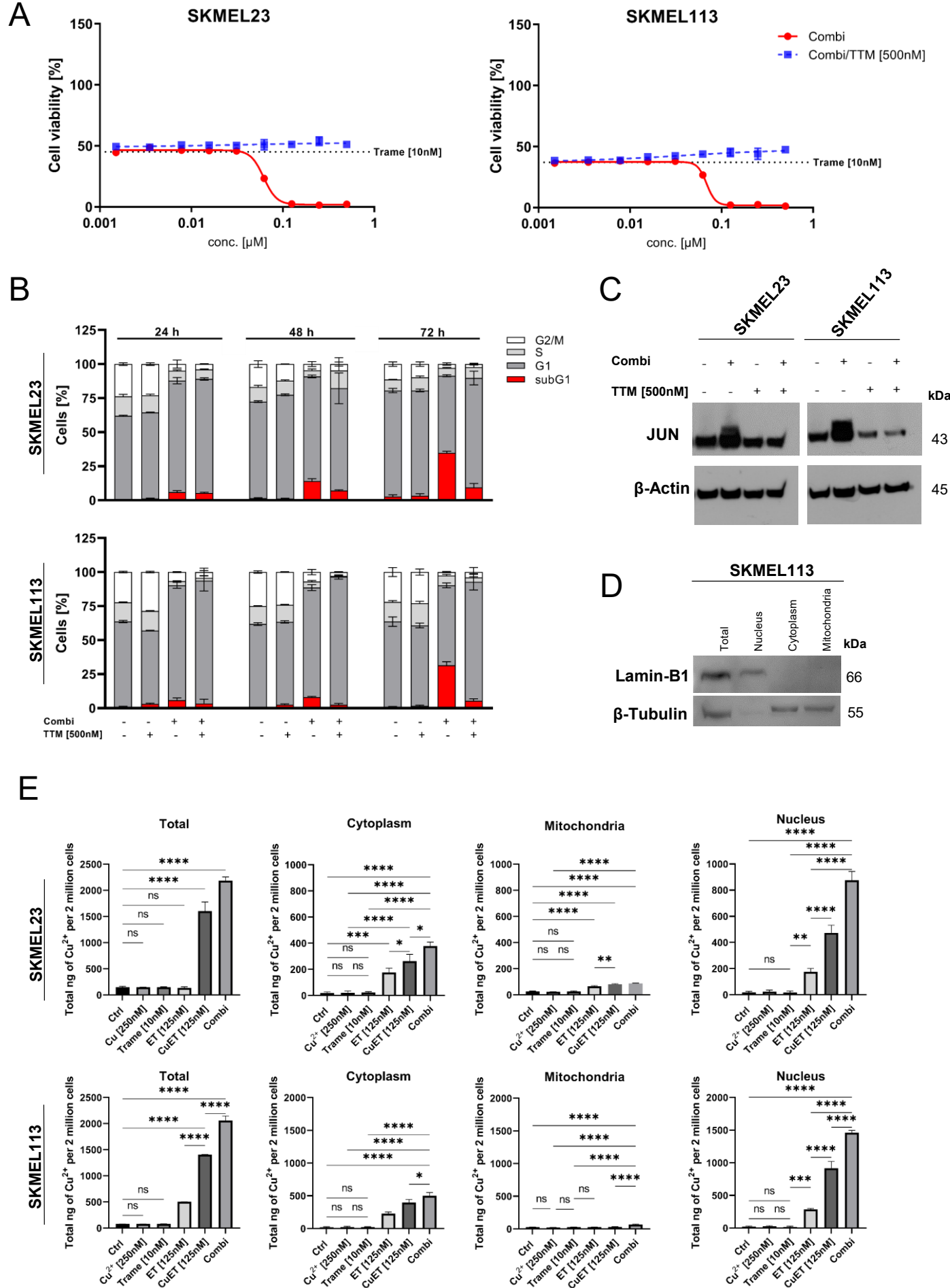

Supplementary Figure 5

Supplement: Supplementary file 1 — Additional file 1. Supplementary Methods, Supplementary Figures, Supplementary Movies, Supplementary Tables. [file 13046_2023_2941_MOESM1_ESM.zip › Supplementary Figure 5.pdf]

A

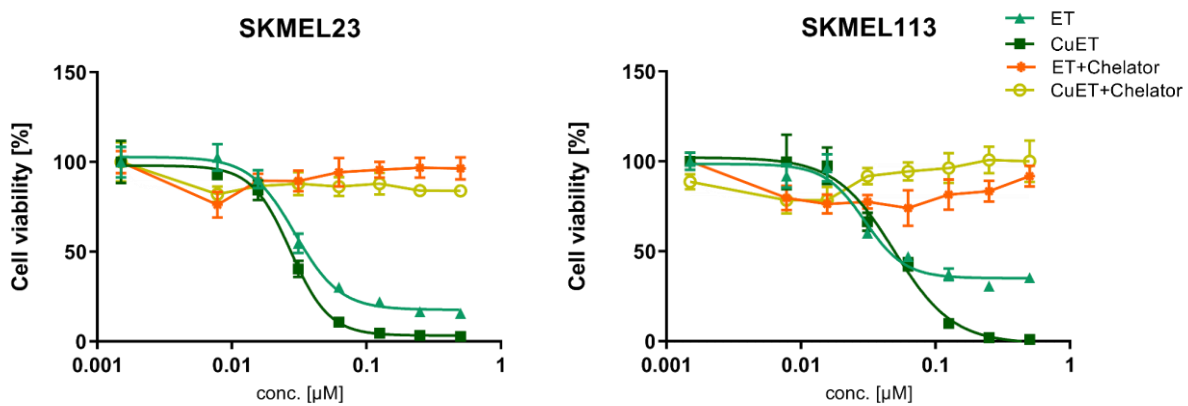

B

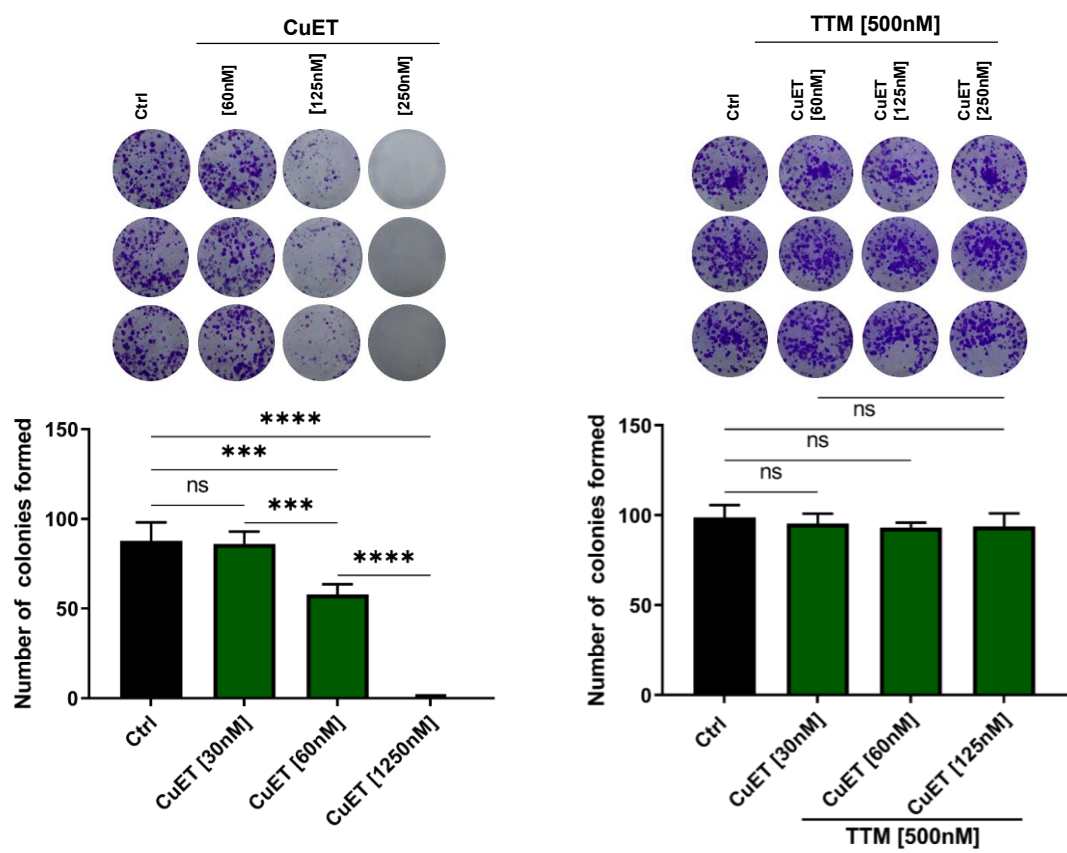

Supplement: Supplementary file 1 — Additional file 1. Supplementary Methods, Supplementary Figures, Supplementary Movies, Supplementary Tables. [file 13046_2023_2941_MOESM1_ESM.zip › Supplementary Figure 6.pdf]

A

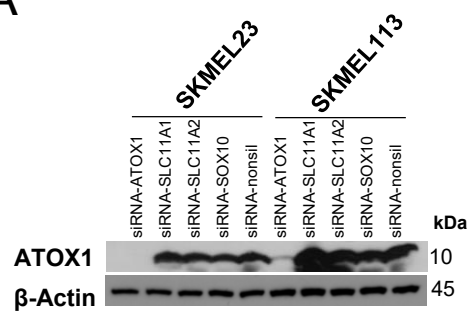

B

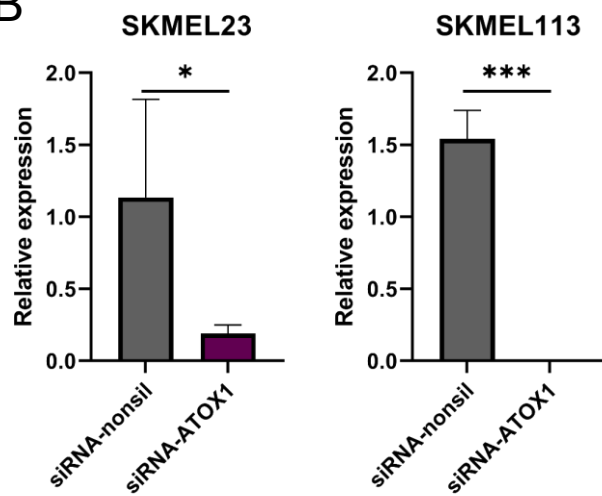

C

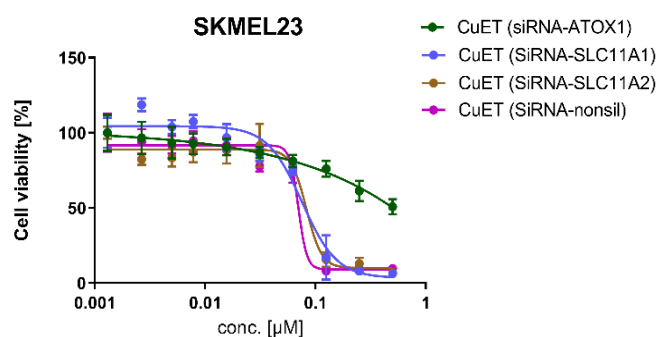

D

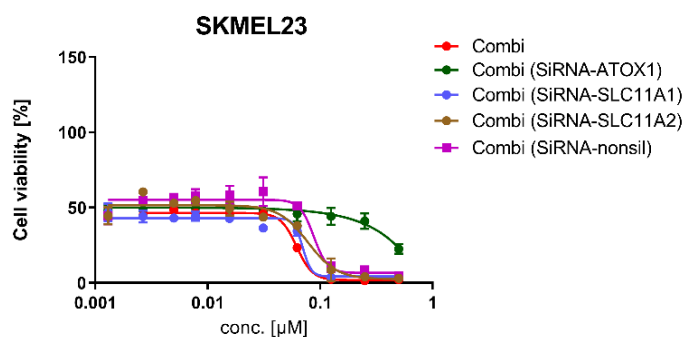

Supplement: Supplementary file 1 — Additional file 1. Supplementary Methods, Supplementary Figures, Supplementary Movies, Supplementary Tables. [file 13046_2023_2941_MOESM1_ESM.zip › Supplementary Figure 7.pdf]

A

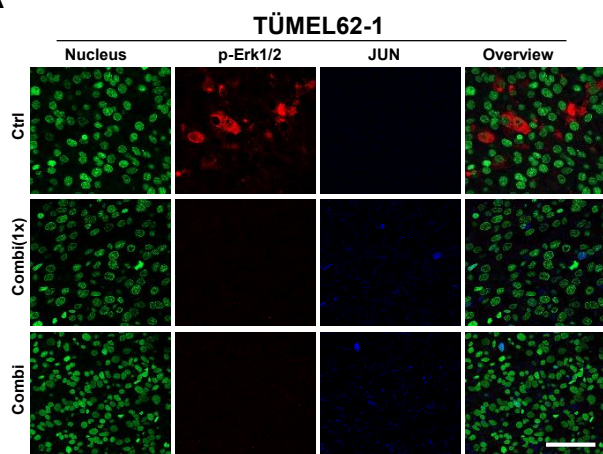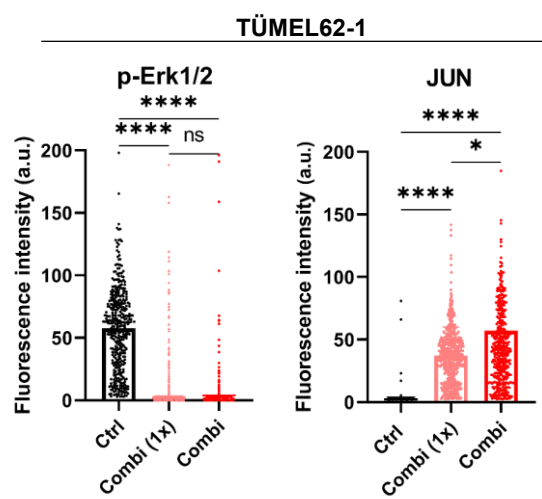

B

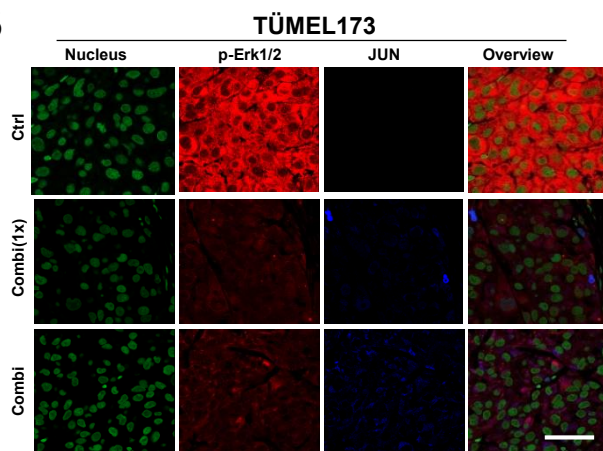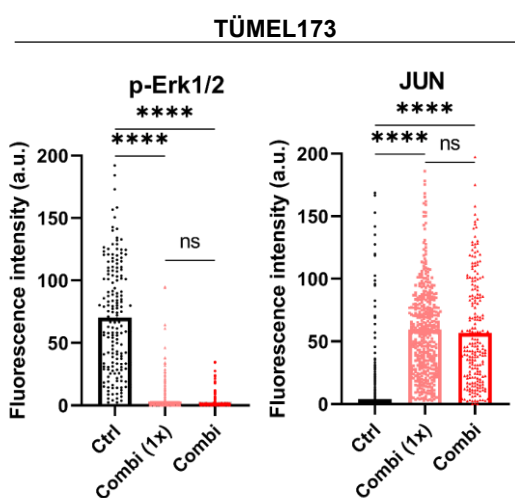

C

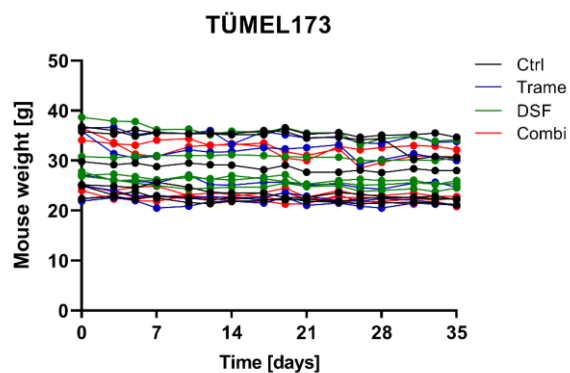

D

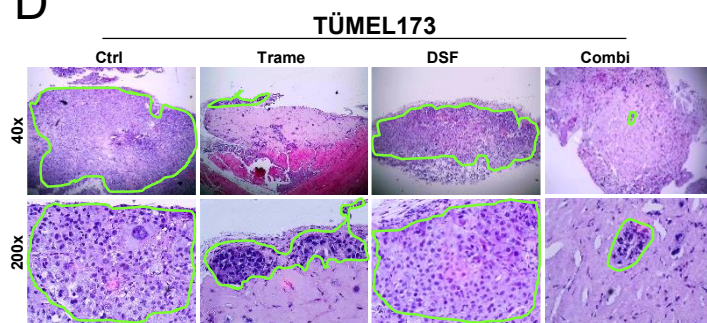

Supplement: Supplementary file 1 — Additional file 1. Supplementary Methods, Supplementary Figures, Supplementary Movies, Supplementary Tables. [file 13046_2023_2941_MOESM1_ESM.zip › Supplementary Figure 9.pdf]
